# A Selective Review of Ceramic, Glass and Glass–Ceramic Protective Coatings: General Properties and Specific Characteristics for Solar Cell Applications

**DOI:** 10.3390/ma16113906

**Published:** 2023-05-23

**Authors:** Rebekah Fraser, Mihaela Girtan

**Affiliations:** 1 P2i Ltd., 127 Olympic Avenue, Milton Park, Oxfordshire OX14 4SA, UK; 2 Photonics Laboratory, (LPhiA) E.A. 4464, SFR Matrix, Faculté des Sciences, Université d’Angers, 2 Bd Lavoisier, 49000 Angers, France

**Keywords:** ceramic, glass, glass–ceramic, barrier coating, protective coating, transparent, photovoltaic, solar cells

## Abstract

A review on ceramics, glasses and glass–ceramics as thin film protective coatings for solar cells is given. The different preparation techniques and the physical and chemical properties are presented in a comparative way. This study is useful for technologies involving solar cells and solar panel cell development at the industrial scale, because protective coatings and encapsulation play a major role in increasing the lifetime of solar panels and environmental protection. The aim of this review article is to give a summary of existing ceramic, glass, and glass–ceramic protective coatings and how they apply to solar cell technology: silicon, organic or perovskite cells. Moreover, some of these ceramic, glass or glass–ceramic layers were found to have dual functionality, such as providing anti-reflectivity or scratch resistance to give a two-fold improvement to the lifetime and efficiency of the solar cell.

## 1. Introduction

Different kinds of materials, including ceramics, glasses and glass–ceramics, have been adopted as protective encapsulation layers for semi-conducting electronic devices such as photovoltaics. Functional ceramics, glasses, and glass–ceramics are those ones that are designed to have specific qualities and perform specific functions.

Glasses are ionic solids with an amorphous network structure; the inclusion of oxides during manufacture prevents crystallisation. They are generally transparent, chemically resistant, durable, and can be easily formed into specific structures during fabrication.

Ceramics are inorganic materials, either crystalline, semi-crystalline or non-crystalline, that have been hardened by heating. They are typically hard, insoluble, stable and resistant to corrosion [1].

Glass–ceramics, a subset of the above, are durable polycrystalline materials formed by controlled partial crystallisation embedded in an amorphous glass matrix [2]. They are typically formed from oxides, such as SiO_2_, Na_2_O, or B_2_O_3_, and they must contain a crystalline phase and a residual glass phase to be classed as a glass–ceramic [3]. Depending on the specific structure, they can appear opaque, semi-transparent or transparent. Maintaining the transparency of glass–ceramics is a challenge in the industry, as the transparency is strongly influenced by the crystal grain size; the larger the crystal size, the more light scattering can occur, and the lower the transparency of the coating [4]. “Activated” glass–ceramics are two-phase systems comprising a glass phase and a crystalline phase doped with ions, such as rare earth metals. The general properties of ceramics, glasses and glass–ceramics have been summarised in Table 1.

The specific properties of ceramics, glasses or glass–ceramics are determined by their structure [6]. Ceramics have a variety of grain sizes, shapes, grain boundaries, and phase boundaries which affect their processing and properties. As a result, the final ceramic characteristics, such as density and hardness, are strongly influenced by the ceramic microstructure [5]. They have a wide range of controllable properties, including but not limited to corrosion resistance, electrical conductivity, mechanical strength, surface functionality (surface energy, catalytic activity, biocompatibility, etc.), and optical properties. These characteristics can be selected by control of the precursor materials, deposition methods and post-deposition treatments.

Glasses and transparent glass–ceramics are typically cheaper and easier to manufacture than transparent ceramics, which facilitates transparency requiring applications. Glass–ceramics can be formed by the heat treatment of the glass during fabrication to induce crystallisation, although this often requires a nucleation agent for initiation. By careful control of the heat treatment temperature and duration, the crystal structure, size and proportion to the amorphous matrix can be controlled, allowing specific optimisation of the glass–ceramics properties [7]. There has been over 60 years of research and development of glass–ceramics, and recently, the focus has been on improving their mechanical properties, thermal shock resistance, transparency, conductivity and optical properties. Transparent glass–ceramics doped with rare-earth ions can exhibit a high luminescence efficiency or radiation downshifting, and as such, they have found application in the photonics or photovoltaic industries [8,9].

In many cases, the desired property is only required on the surface and not as part of the bulk material so a specifically functionalised layer can be applied over the surface. This allows the wider use of materials which cannot otherwise form the bulk of the structure by using multilayers or a top coating to achieve the desired effect, such as self-cleaning or anti-fouling [10]. Ideally, such top coatings should have a high durability and be conformal to 3D structures for optimal substrate coverage, but this is more difficult to achieve. Many coatings struggle in terms of hardness and wear resistance or can only be applied to planar structures due to their deposition methods. An appropriate hydrophobic coating can improve the self-cleaning, anti-icing and anti-frosting qualities; however, it can be difficult to strike the right balance, as typically, an increase in surface roughness (which is commonly used to increase the hydrophobicity) leads to a decrease in light transmission due to Mie scattering, making the coating less appropriate for certain applications [11].

**Figure 1:**
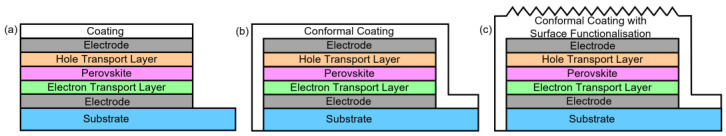
Schematic of protective coating on a solar cell, showing (**a**) a flat coating over the top of the device, (**b**) a conformal coating over the device, and (**c**) surface functionalisation to enhance the protective effect.

The primary focus of this review is on the application of functional ceramics, glasses and glass–ceramics as protective layers, specifically with regard to optical and photovoltaic applications. Solar cells can be exposed to harsh environments, and they experience damage by physical ablation by dust, water damage by rain or humidity exposure, chemical damage by acid rain or salt spray to name but a few causes of degradation and efficiency losses. As such, they require protective coatings to extend their lifetime and maintain their power conversion efficiency (PCE), which often comes in the form of a glass encapsulant or polymeric coating. However, these have their own drawbacks such as weight, fragility or discolouration over time, so careful selection is required. A protective coating or encapsulant can be applied to a photovoltaic cell in three primary ways: a flat rigid coating over the top of the cell, a conformal coating that covers the three-dimensional structure of the cell, and as a surface treatment to enhance the protective nature of an existing coating, as shown in Figure 1.

Perovskite solar cells (PSCs) are some of the most promising emerging candidates for future solar cell development, with reported PCEs rising from less than 4% in 2010 up to 25.2% in 2021 [12,13]. These types of cells provide a viable alternative to the current standard silicon-based cells, which have a long energy payback time and can be expensive to produce [14]. The potential for large-scale deployment is held back by the stability of the cells: only one year of device stability for PSCs versus 25 years for silicon-based cells [15]. Their longevity can be improved adequate protection and shielding from the environment, and so they require durable and reliable encapsulation, making this a rapidly increasing research field [16]. The object of this review is to assess the latest developments in the fields of ceramics, glasses and glass–ceramics in terms of their potential applicability as protective barrier coatings for solar cells.

## 2. Literature Review

The information contained herein was sourced primarily from articles from the period 2018 to 2023 which featured ceramic, glass or glass–ceramic protective coatings. Special attention was paid to those which either specifically stated that they could be applied as protective coatings for solar cells or those that demonstrated transparency. Due to the extremely high volume of research in this area, not all relevant articles may have been mentioned, but a sincere attempt was made to obtain as representative an overview as possible.

### 2.1. General Properties and Classification

#### 2.1.1. Mechanical Properties

The Young’s modulus and fracture strength of a material depend on the strength of the atomic bonds and the inter-atomic bonding forces. Carbides, nitrides and borides tend to be the strongest single non-oxide ceramics due to their covalent or covalent metallic bonding. The majority of boride ceramics have a high melting point, oxidation resistance and mechanical strength, and low mass density and coefficient of thermal expansion (CTE), which make them suitable for many applications which require good durability [17]. The strength, hardness and elastic modulus of ceramics are highly linked to the porosity, and these properties can vary substantially in response to slight changes in porosity. Generally, the strength and hardness of the ceramic exponentially decrease as the porosity increases. The chemical resistance and bioactivity of advanced ceramic coatings are also influenced strongly by the porosity and pore size, with the biological and chemical activities tending to increase with an increase in porosity. Advanced ceramics which are designed to have a specific bioactivity or chemical activity usually require a specific porosity and pore size to maintain the effect, whilst ceramics designed for strength require the porosity to be low [17]. The required mechanical properties are achievable by control of the porosity during the deposition phase and by the addition of organic groups to the structure [18]. The roughness and surface morphology of the material can also influence the wear resistance and durability of the coating.

#### 2.1.2. Optical Properties

Ceramic materials can be transparent, translucent, or opaque depending on their microstructure, particularly the structural features that cause the diffusion of incident light. The optical transparency of a coating relies on three key factors: the refraction, the deflection and the transmission of light. Absorption in the visible region of the spectrum can arise from pores and point defects of the crystal lattice, so the lattice purity must be carefully controlled to maintain the desired level of transparency [17]. When refractive indices are discontinuous, the grain boundaries can cause light scattering, so matching the refractive indices of the different phases or structures can reduce the amount of scattering and increase the transparency. Light scattering through mixed materials is generally expressed by Equation (1):

(1) $$\tau \propto {\left(\frac{∆n}{\overset{-}{n}}\right)}^{2}\frac{L^{4}}{\lambda^{3}}$$

where:

∆n is the difference between the refractive indices of the glass and crystal phases;

$\overset{-}{n}$ is the average of these refractive indices;

L is the size of the crystal particles;

λ is the wavelength.

Transparency can generally be maintained with a crystallite size of around 30 nm or less, and scattering can also be reduced by limiting the number of scattering features as well as the size [7]. Glass–ceramic manufacture must fulfil two criteria to maintain the transparency: the crystalline phase refractive index must match the glass phase, and the crystal size must be small to reduce scattering [4]. Full transparency depends on the high in-line transmission of light through a material; otherwise, it will appear translucent or fully opaque. This can be controlled by careful selection of the deposition methods and conditions and the post-coating treatment conditions.

#### 2.1.3. Classification

Functional glasses and glass–ceramics are generally classified based on their composition, processing methods, the resultant properties, and their application. Composition of the coating generally falls into two primary compositions: oxides and non-oxides. Oxide ceramics are non-metallic, inorganic compounds such as alumina (Al_2_O_3_), zirconia (ZrO_2_), silica (SiO_2_) and magnesia (MgO). Oxide ceramic coatings tend to possess high electrical resistivity, wear resistance and oxidation resistance. Non-oxide ceramics, such as carbides, borides, nitrides and silicides, tend to fare better in terms of thermal shock resistance and fracture resilience [19]. The processing category defines the preparation of the glass or ceramic and the physical process used in its formation, whether that is sol–gel, chemical vapour deposition (CVD), physical vapour deposition (PVD), sputtering, melt quenching, etc. or whether the material requires doping with rare-earth metals, a specific crystalline structure or the incorporation of metal nanoparticles to achieve the desired properties. Possibly the most useful of the classifications, the properties of the coating can be used to separate the different types, such as thermal, physical, optical, photonic, electrical, mechanical, chemical etc., and they can be further subdivided within those categories into the more specific effect, such as the CTE, density, or conductivity. The coating applications depend heavily on the properties [6]. For example, a glass–ceramic applied as an insulating coating would require a low conductivity, or applied as an environmentally protective coating, it would require high chemical inertness and low water permeability. Optical applications require high transparency, low reflectivity or a specific refractive index. Encapsulants require mechanical strength and durability to protect the substrate as well as any other qualities, such as transparency and chemical resistance, depending on the specific application.

#### 2.1.4. Types of Deposition

There are many deposition methods used to form ceramic, glass and glass–ceramic coatings, Table 2. There are benefits and drawbacks to each method, and the choice of technique will be influenced strongly by the substrate, application, type of coating desired and precursor materials.

Historically, CGDS was developed for spraying metallic powders and metallic–ceramic composites, with the ceramic included to improve the mechanical strength of the coating. With control of the deposition conditions and careful selection of the feedstock, the obtained coatings can have enhanced strength, durability, electrical resistance, chemical resistance, transparency, and robust thermal properties, which make these coatings appropriate for a wide range of applications. Depending on the types of powders selected, these coatings can have optimised optoelectronic, photocatalytic, or biocompatible properties, or they can have improved mechanical properties such as hardness, wear resistance, thermal insulation, electrical insulation or corrosion resistance. The challenges found in these coating types were typically in the cohesion and adhesion of the coating to the substrate. Transparent hydrophobic coatings were deposited by vacuum cold spray, as discussed by Li et al., who used the method to deposit a fluoro-functionalised Al_2_O_3_/SiO_2_ coating [21]. The cold spray method gave coatings with properties highly dependent on the precursor materials. To form transparent hydrophobic coatings, Li et al. selected sub-micrometre Al_2_O_3_ combined with a porous SiO_2_ aerogel. The Al_2_O_3_ was modified with fluorosilane, first by hydrolysis of the fluorosilane and Al_2_O_3_ then a dehydration reaction to form the fluoro-functionalised Al_2_O_3_ to impart hydrophobicity. The precursor choice and deposition conditions influenced the surface roughness of the deposited coating and thus influenced the coating properties. The coatings with a higher Al_2_O_3_ to SiO_2_ ratio (5:1 and 4:1) showed the best transparency, at around 90% transmission, and this dropped to 70–80% for the coatings with the lower ratio (2:1). The surface roughness peaked at 0.294 ± 0.005 µm for the Al_2_O_3_ to SiO_2_ ratio of 3:1. This was selected as the best coating, as it gave a balance of high proportion of the fluorinated component, and so a high contact angle, enhanced by good roughness, and relatively high transparency at >80% transmission. The fluorosilane-modified Al_2_O_3_-only coating was relatively flat and dense, so it had a comparatively poor hydrophobicity but high transparency [21].

Cold spray and aerosol deposition methods, as discussed by Akedo, accelerated microparticles into a beam at high velocities, which then physically impacted the target substrate and formed a coating by room temperature impact consolidation (RTIC) [33]. The films formed in this way tended to be dense and well adhered to the substate with a controlled thickness and microstructure. Aerosol depositions are a subset of gas deposition, in which a carrier gas suspended the precursor microparticles and the suspension was then sprayed onto the substrate under reduced pressure. Films formed using these methods did not require sintering, and as there was no need to heat the substrate to high temperatures, they could be deposited on a wider variety of substrates. Additionally, Al_2_O_3_ and aluminium nitride films deposited in this manner had a high transparency (98%) when deposited up to 5 µm. It was thought that this was due to the suppression of optical scattering because of the smaller crystal size. This feature of high transparency, combined with their high volume resistivity, high electrical breakdown strength and toughness made them extremely promising candidates as protective coatings or encapsulants for photovoltaic devices. The lack of sintering also could facilitate the deposition on flexible plastic substrates which are coming into greater use in the field of photovoltaics due to their compatibility with emerging ultra-thin transparent electrode technologies [48,49,50]. However, if the grain size deposited was too low, the performance could be lost, and curing at 500–900 °C was required. TiO_2_ layers deposited using aerosol deposition have already been used in the formation of flexible dye-sensitised solar cells (DSSCs). Insulating yttria (Y_2_O_3_) films were deposited which had excellent mechanical strength and hardness, a high volume resistivity, and high breakdown strength, performing better than the bulk material [33]. Using standard methods, these films needed to be treated at 1700 °C after deposition, but that was not required with RTIC methods.

Sol–gels were a cheap and versatile avenue to obtain pure, homogenous ceramic or glass–ceramic coatings with a variety of possible nano- or micro-structures at low sintering temperatures, usually in the realm of 100–600 °C [36]. The sol–gel precursor could be prepared and then deposited by liquid coating methods, such as spray coating, spin coating or dip coating, and this was usually followed by a heating or curing stage. Many different types of ceramics have been formed using the sol–gel method, including Al_2_O_3_, SiO_2_, ZrO_2_ and chromium-based coatings. The sol–gel could be used to fabricate multicomponent materials with strictly controlled compositions, shapes, textures and morphologies. This technique is highly flexible and has even been used to incorporate rare earth dopants for the functionalisation of oxide coatings [18]. Glass–ceramic coatings were very commonly formed using sol–gels or by melt quenching a glass precursor followed by a heat treatment to initiate crystallisation. Silicon oxycarbide (SiOC) glass network and glass–ceramic coatings have been prepared by a variety of methods due to the high processability of the preceramic SiOC sol–gel, such as spin coating, dip coating, spray coating, soaking, melting, brush coating, and thermal CVD [51]. Making the final product from the sol–gel precursor, however, required several different steps. Firstly, the thermoplastic precursors were crosslinked at low temperatures (100–400 °C); then, the organic/inorganic network was thermally converted into glass via pyrolysis (600–1000 °C), which was followed by phase separation (>1200 °C). Continuing the heat treatment to 1400 °C resulted in a greater degree of polycrystalline material formed and a more glass–ceramic-like final structure [52,53]. SnO_2_-SiO_2_ glass–ceramics doped with rare earth elements historically have been prepared by modified chemical vapor deposition (MCVD) or melt quenching, but these methods gave a low concentration of the SnO_2_ component due to SnO_2_ decomposition to more volatile species, e.g., oxygen and SnO [18]. Conversely, the low-cost, low-energy sol–gels have been exploited to obtain glass–ceramics with a significantly higher percentage of SnO_2_. Glass–ceramic coatings comprising 80SiO_2_-20LaF_3_ doped with Er^3+^ were formed from sol–gel processing [36]. These coatings were homogenous, crack-free, highly transparent and well adhered, which made them very appropriate for photovoltaic applications. The deposition method required a short heat treatment step of one minute at 550 °C, which unfortunately made it a less suitable coating method for temperature-sensitive substrates. Structurally, these oxyfluoride glass–ceramics comprised fluoride nano-crystals in a glass matrix, and the inclusion of a rare-earth dopant increased the luminescence efficiency compared to the analogous oxide glass. An approximately 18 wt% LaF_3_ crystal fraction was found for this glass–ceramic, which was very high compared to the standards for this sol–gel or melt-quench processing [36]. Other rare-earth elements could be used as dopants, such as Nd^3+^ [38,54]. These coatings were heat treated at 350 °C after dip coating of the sol–gel nanoparticle suspension, giving different coating properties on heating to different temperatures. As the temperature of the heating step increased, the thickness of the resulting coating decreased as shrinkage occurred. Conversely, the refractive index of the coating increased for the coatings heat treated at higher temperatures. This was thought to be caused by condensation of the SiO_2_ network and loss of residual organics and an increase in the LaF_3_ crystal fraction [54].

In the formation of oxide ceramic coatings, plasma spraying, particularly atmospheric plasma spraying (APS), was one of the most extensively used methods of thermal spray deposition. The feedstock was usually a mechanically ground, crushed or fused powder, such as Al_2_O_3_, Cr_2_O_3_ and TiO_2_, with a typical particle size of around 15–45 µm. The properties of single-oxide coatings were improved by the addition of a second or third component to the feedstock. Ternary blends were reported by Grimm et al. in 2020 by APS [24]. In these three component mixtures, the ratio of the components had a significant influence on the coating properties. Regardless of the ratio of the feedstock, all ternary blend oxide coatings were well adhered to the substrate as compared to the single-oxide counterparts. As expected, the roughness of the three-component blend coatings sat between the roughness of the smoothest single oxide coating (Cr_2_O_3_, 33 ± 2 µm) and the roughest (TiO_x_, 70 ± 2 µm). The porosity was also found to be variable; the TiO_x_ single-oxide coating was the densest and the Al_2_O_3_ was the least dense, and the three component blends had porosities between these values. In general, the ternary blends were softer than the single oxide counterpart, with the exception of the blend with the highest proportion of Cr_2_O_3_, and the wear resistance was greatest for the coatings containing the highest proportion of Cr_2_O_3_ [24].

High-intensity laser irradiation-assisted CVD has been used to rapidly deposit well-ordered ceramic coatings [44]. Structural control using this method could also be applied to the deposition of more conventional ceramics such as Al_2_O_3_, TiO_2_, Al_2_TiO_5_, BaAl_12_O_19_, BaTiO_3_, YBa_2_Cu_3_O_7_-δ, and CeO_2_ to impart specific functional properties to the coating. The films grown in this way, as reported by Ito, were self-oriented and, due to the control of the crystal structure, were transparent [44]. The laser-assisted CVD was able to achieve the deposition of the specific α-Al_2_O_3_ polymorph, and depending on the laser parameters, anatase or rutile TiO_2_ films could be selectively deposited. Porous, feather-like β-Al_2_TiO_5_ and transparent, conductive BaAl_11_O_17_ could also be deposited with this method. As an alternative to single crystals, the epitaxial growth of transparent layers composed of BaTiO_3_, BaTi_2_O_5_, and the high-temperature superconductor YBa_2_Cu_3_O_7_−δ was reported [44]. Plasma-assisted CVD was used to deposit TiAlCN coatings with variable properties depending on the blend ratio of the precursor feedstock. For example, the TiAlCN coating with the lowest proportion of Al had the highest hardness and roughness values, and comparison coatings with higher proportions of Al were softer and smoother [45].

Each of these commonly used coating deposition methods had positive and negative points in terms of the coating’s potential application as a protective layer for solar cells. Wet, sol–gel-based methods were very useful on the small lab scale, as they had a high degree of versatility and allowed the deposition of many different coating types and chemistries. However, when taking production scales and requirements into account, other methods became more desirable. Spraying-based techniques and vapour deposition methods were more applicable on large scales as they provided better coating conformality over 3D substrate architectures and were suitable for large-scale deposition. Additionally, care must also be taken to ensure that the deposition method was compatible with the type of solar cell used, as temperature-sensitive flexible substrates or cell components could be damaged during a sintering process or high-temperature deposition. The coating type desired, the substrate components and structure, the scale and throughput required, and whether a batch or inline process was most appropriate all influence the method selection and the determination of which method is the “best” one.

### 2.2. Ceramic, Glass and Glass–Ceramic Applications as Protective Coatings

#### 2.2.1. Ceramics

Ramesh et al. described a functionally graded ceramic coating, comprising yttria-stabilised zirconia (YSZ) and Al_2_O_3_, which acted as a thermal barrier coating deposited on steel by an APS process [55]. Two distinct coating compositions were found depending on the carrier gas concentration, giving an improvement in insulation performance, wear and scratch resistance for the lower carrier gas flow. This was due to the increased percentage of YSZ in the blend using the lower carrier gas flow; YSZ had a higher thermal shock resistance, higher CTE and lower thermal conductivity than the Al_2_O_3_. However, YSZ alone can be unstable and subject to corrosion, so the inclusion of Al_2_O_3_ imparted some mechanical strength and stability to the coating. The decreased Al_2_O_3_ present in the coating formed from the lower carrier gas flow resulted in a less dense, rougher final coating than the higher Al_2_O_3_ ratio counterpart, although the difference was small (6.25 ± 0.02 µm to 6.31 ± 0.05 µm).

**Figure 2:**
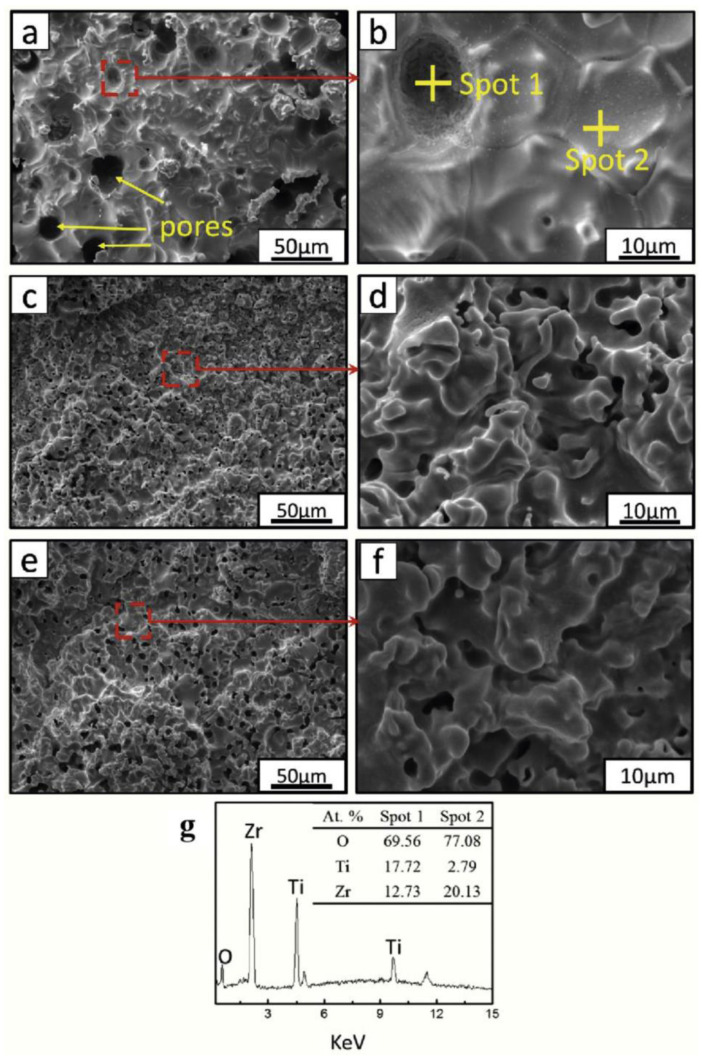
SEM images of surface morphology of spraying coating after ablation for different time periods. (**a**,**b**): 120 s; (**c**,**d**): 180 s; (**e**,**f**): 240 s; (**g**): Energy-dispersive spectrometer (EDS) of Spot 1 and Spot 2 in (**b**). Reprinted from [25], with permission from Elsevier.

A comparison study by Newkirk et al. compared ceramic coatings such as TiO_2_ and ZrO_2_/SiO_2_/ZrO_2_/SiO_2_ with various other surface coatings and treatments, such as porous SiO_2_, fluoropolymers, silane-functionalised and roughened glass against uncoated glass or plastic substrates [56]. The aim was to compare the wear resistance of the different transparent coatings used to protect photovoltaic devices. After the slurry abrasion test, the uncoated polymer substrate had discoloured most and had the biggest loss in light transmission, as was expected. The rest of the samples performed well with little change in transmission or colour, although the porous SiO_2_ and etched glass treatment showed some minor variance over the course of the test. All samples showed some change in contact angle, but the silane-functionalised surface had the most significant change, going from >100° to <40°. This was not due to changes in the roughness, as they remained relatively consistent over the abrasion testing, and the roughness increased in the untreated polymer with some variance to the porous SiO_2_. Similar results were seen for the dry dust test. The TiO_2_ and multilayered ZrO_2_/SiO_2_/ZrO_2_/SiO_2_ stack had greater durability than the other tested coatings, showing more consistency over the course of the test before eventual breakdown and removal of the coating [56]. This study supported the use of ceramic coatings as durable protective coatings for photovoltaic devices. Other ablation-resistant coatings formed of ZrB_2_-SiC-TiSi_2_ have been reported by Li et al. [25]. Two types of coating were deposited to make up the coating system: a silicon carbide inner coating and the ZrB_2_-SiC-TiSi_2_ as an outer coating. The resulting protective coatings were rough and defect free, and they were able to withstand ultra-high temperatures of up to 2230 K for 240 s, although some cracking did appear after 60 s ablation testing, as shown in Figure 2 [25].

Transparent scratch-resistant Yttrium/Sialon (a mix of silicon, aluminium, oxygen and nitrogen) coatings were deposited by Mohamedkhair et al. by pulsed laser deposition [57]. These coatings showed a high transparency and a roughness which was tuneable depending on the substrate temperature; a cooler substrate led to a rougher coating, with RMS values of 16 nm for room temperature deposition compared to <10 nm for 500 °C substrates [57]. Additionally, the room temperature substrate showed the highest transparency at just over 90%. However, the 500 °C substrate showed a significant improvement in scratch resistance [57]. This unfortunately could rule out several more temperature-sensitive substrates, but there could be benefit in the application of these coatings to temperature-stable coatings which required a transparent scratchproof coating or depositing at room temperature, where scratch resistance was not required.

**Figure 3:**
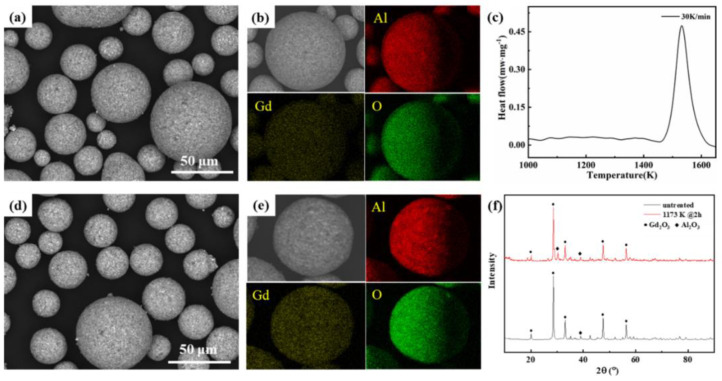
(**a**) SEM image of granulated powders (the lower–left corner shows the particle size distribution of granulated powders); (**b**) the morphology of the single granule and related element distribution; (**c**) DSC curve of Al_2_O_3_/Gd_2_O_3_ granulated powders at the heating rate of 30 K/min; (**d**) SEM image of granulated powders sintered at 1173 K for 2 h; (**e**) the morphology of the single granule sintered at 1173 K for 2 h and related element distribution; (**f**) XRD patterns of Al_2_O_3_/Gd_2_O_3_ granulated powder. Reprinted with permission from [28], Creative Commons License 4.0.

As a strong material, Al_2_O_3_ has long been the typical benchmark for wear-resistant oxide ceramic coatings, and TiB_2_ has a high melting point, good hardness, high elastic modulus and a good resistance to oxidation. Together, these components could be combined to form a wear-resistant Al_2_O_3_-TiB_2_-TiC coating, as documented by Li et al. [47]. Coating compositions of 10%, 30% and 50% Al_2_O_3_ were tested, and it was found that the coating with the lowest content of Al_2_O_3_ was the hardest, but the optimal level for increased wear resistance was a blend containing 30% Al_2_O_3._ The higher hardness for the 10% Al_2_O_3_ blend could be due to the faster solidification of the TiB_2_ because of its higher melting point and poor wettability between the components. The 30% blend was potentially closer to a eutectic mixture and a more uniform coating, resulting in better wear resistance. Above 30% grain boundary cracking occurred due to the brittleness of the Al_2_O_3_ [47]. An amorphous ceramic coating comprising Al_2_O_3_-GdAlO_3_ (GAP) was prepared by APS from sprayable powder precursors [28]. This coating, as described by Qiang et al., had an excellent microstructure uniformity and mechanical performance, as shown in Figure 3. Additionally, the amorphous Al_2_O_3_ ceramic coatings showed greater plasticity, crack suppression and wear resistance compared to standard Al_2_O_3_ and Al_2_O_3-_Cr_2_O_3_ coatings.

**Figure 4:**
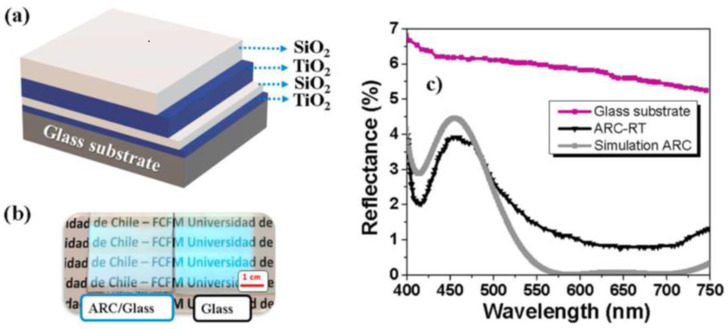
(**a**) Schematics of the used multilayer system. (**b**) Verified anti-reflective effect by optical inspection and (**c**) reflectance versus wavelength for the glass substrate and the ARC-RT sample. Moreover, the results of the numerical simulation are shown for comparison. Reprinted from [58], with permission from Elsevier.

Anti-reflective coatings were formed by alternating layers of TiO_2_/SiO_2,_ as reported by Zambrano et al., as shown in Figure 4 [58]. The effect of a durable anti-reflective coating is twofold: an improvement in the light collection efficiency due to the reduced reflection and protection by the hard coating of the underlying cell from damage, resulting in a longer-lived, higher efficiency solar cell.

These coatings had a dense, homogenous anatase microstructure, with an approximate grain size of 50 nm after annealing at 400 °C for one hour. This annealing step was necessary to induce anatase TiO_2_ formation to increase the coating density and reduce the surface roughness. Smaller grain size and reduced surface roughness caused less light scattering and therefore a higher transmission efficiency, so they performed better as anti-reflective coatings overall. The alternating multilayer coating system had a reflectivity of <3% [58]. ZrO_x_ doping was used to improve the durability of anti-reflective TiO_2_/SiO_2_ layers for photovoltaics, as described by Zambrano-Mera et al. [59]. The coatings were deposited by magnetron sputtering and formed Zr–O–Si bonds which enhanced the crystallinity and density of the coatings and thus improved their durability. The doping was applied in the final layer of a multilayer coating system of thin TiO_2_/SiO_2_/Si–Zr–X layers. Thermal annealing at 400 °C for one hour resulted in an increase in refractive index for all of the coating iterations but had a greater effect on coatings with a lower dopant concentration [59]. Additionally, the coatings showed an increased reflection in the UV region, which could be beneficial in reducing the breakdown of polymers and organic compounds in solar cells. The concentration of Zr in the SiO_2_ regions affected the hardness and Young’s modulus of the coating. With a small dopant concentration, the coating hardness increased, but a further increase showed a reduction. This was attributed to the generation of Zr–O and Zr–Si oxides, which increased the density of the SiO_2_ top layer. Further increasing the Zr concentration resulted in Si–Zr–O bonds forming, increasing the elasticity and reducing the hardness [59]. This gave the coating a tuneable hardness and elasticity, making these very promising coating systems with a great deal of potential for further applications, although the requirement of an annealing step was not ideal for temperature-sensitive substrates. Zr incorporation has also been used elsewhere to improve the mechanical properties of a coating, such as that described by Chen et al. [60]. Although the primary focus was anti-fouling and oil repellency, the wet solution deposited epoxy-ZrO_2_ coatings also proved to have a high wear resistance, high UV and chemical resistance, good adhesion and good flexibility. This made them incredibly promising candidates as protective coatings for photovoltaics. Although ZrO_2_ has previously been used as a ceramic coating, as well as Al_2_O_3_ and TiO_2_, it has been found that a ZrO_2_ coating doped with a small amount of TiO_2_ showed improved durability and transparency as compared to the single-component coating. At just 3 wt% TiO_2_ dopant concentration, the ZrO_2_ coatings had a low porosity, high density, high fracture toughness and high hardness [41].

Many anti-reflective coatings have been designed to include a degree of radiation downshifting to increase the amount of incident usable light [8,9,61,62]. Coatings have been designed for windows which reflect IR radiation but were transparent to visible radiation. These coatings were multilayer stacks comprising TiO_2_/Ag/TiO_2_ and have been protected by sputtered ceramic materials such as ZnO, Al-doped ZnO or Al_2_O_3_ [39,50]. Van Zele et al. describe low-temperature (<100 °C) spin-coated or roll-coated sol–gel deposited SiO_2_ layers as a protective coating to the stack or substrate. These coatings had good adhesion, high scratch resistance and high transparency, making them suitable candidates for photovoltaic applications [39]. Thin layers of Al_2_O_3_ and SiO_2_ were deposited by ALD on organic solar cells as a dual functional anti-reflective coating and permeation barrier. These coatings successfully protected the underlying cell from moisture-induced degradation. When exposed to 85 °C and 85% RH for 500 h, the unencapsulated comparison cell showed a 16% decrease in PCE compared to the protected cell. The coating was shown to reduce the reflectance of the device significantly and increase the PCE from 21.1 ± 0.2% to 27.4 ± 0.3%. Significantly, the coating had a bending radius of 15 mm with no loss in performance up to 1000 bend tests, allowing a high degree of flexibility in the cell, opening up flexible solar cell applications [63]. These layers appeared to be extremely promising as barrier coatings for solar cells and certainly warranted further investigation. Exposure to harsher environments and increased test duration would probe the limits of these types of coatings. Furthermore, the deposition method was applicable for scale up to production levels requiring no high temperatures, which was an added benefit, as fragile substrates were not eliminated.

Corrosion protection barriers have been formed from ceramic coatings, often containing SiO_2_, TiO_2_, Al_2_O_3_, Cr_2_O_3_, or Y_2_O_3_ due to the properties of these materials. Composite Al_2_O_3_-TiO_2_ oxide ceramic coatings were described by Zavareh et al. [26]. These Al_2_O_3_-TiO_2_ coatings were deposited by thermal spray and had a low porosity, high mechanical strength, high oxidation resistance and high electrical barrier performance [26]. SiO_2_-based polymer ceramic composite barrier coatings were formed from perhydropolysilazane (PHPS) precursors to give excellent permeation barriers against water and oxygen [64]. These coatings, as reported by Channa et al., were cured with a relatively low heat (65–100 °C) compared to other ceramic coatings or by exposure to UV irradiation, so they were applicable to substrates which could not tolerate the extreme heating that would otherwise be required. This method was used to produce dense, defect-free SiO_2_ films which were specifically evaluated as barrier coatings for organic solar cells due to the low permeation and high transparency. As well as being transparent and impermeable at relatively low thicknesses, these coatings were reported to have low shrinkage and crack resistance. PHPS films obtained in this way fully satisfied the requirements for organic photovoltaic device encapsulants and were processable at room temperature ambient conditions, making them an exciting avenue for future barrier coating developments and applications.

**Figure 5:**
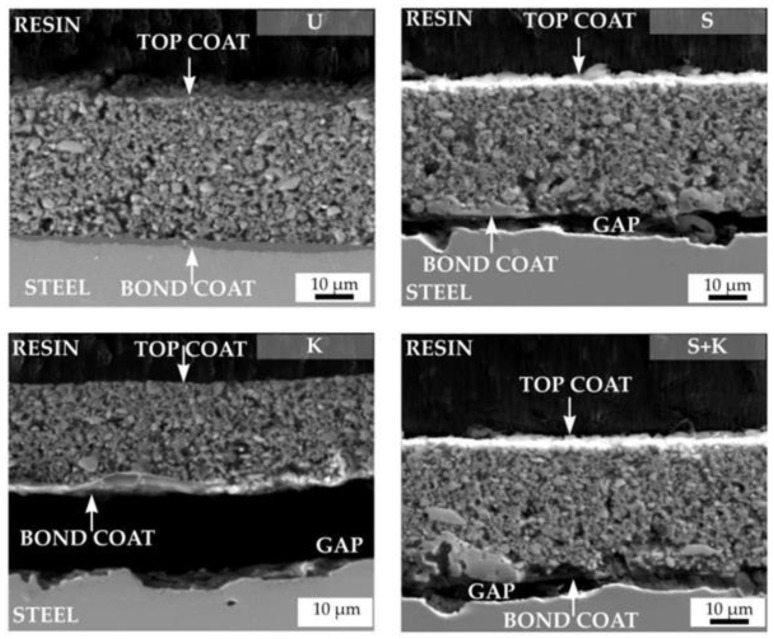
Cross-sections of D2-PP coatings after oxidation tests. The applied cleaning procedure is indicated by the abbreviation in the upper right corner of each figure (U—ultrasonic cleaning, K—chemical etching with Kroll’s reagent, S—sandblasting, S + K—sandblasting + chemical etching with Kroll’s reagent). Reprinted with permission from [42], Creative Commons License 4.0.

Ji et al. reported the process of using a pore sealing step on electrolytically deposited γ-Al_2_O_3_ layers when the precursor was aluminium isopropoxide [65]. The pore-sealing step comprised dip coating the Al_2_O_3_ coated substrates in an epoxy resin to give improved corrosion protection as determined by electrochemical impedance spectroscopy (EIS). The γ-Al_2_O_3_ performed better than the comparison MgAl_2_O_4_ deposited using an aluminium nitrate precursor [65]. Polymer-derived ceramic (PDC) coatings were deposited by spray coating, and as described by Parchovianský et al., they gave dense, well-adhered coatings without delamination or cracking even at very high temperatures, although a bond coat/primer layer and optimised pretreatment process were required in the described research, as shown in Figure 5. The Al_2_O_3_-Y_2_O_3_-ZrO_2_ (AYZ) PDC coatings provided excellent oxidation protection to the substrate [42].

Enhanced corrosion protection was also achieved by incorporating TiO_2_ nanoparticles in a Al_2_O_3_/ZnO phosphate ceramic coating [66]. The phosphate ceramic coating was formed by powder coating after finely grinding the precursors to distribute the TiO_2_ nanoparticles in the ceramic. The nanoparticle addition raised the curing temperature of the ceramic and slowed the reaction time, resulting in a denser, more tightly packed coating with a lower number of voids. The longer reaction time also allowed the formation of AlPO_4_, which acted as a bonded phase and improved the anti-corrosion performance of the coating. Additionally, the water contact angle increased with the addition of the nanoparticles, from <100° to >120°, which also assisted in corrosion protection by displacing water from the surface. Increasing the concentration of TiO_2_ nanoparticles up to 9 wt% led to an increase in the resistance of the coating as measured by EIS [66]. The superior corrosion protection of these coatings made them suitable barrier coatings, and the hydrophobicity was an additional benefit.

Optically transparent ceramic crystalline–amorphous (AlN/SiO_2_, AlN/Al_2_O_3_) and amorphous–amorphous (TiO_2_/SiO_2_) multilayered coatings were deposited by magnetron sputtering to give multifunctional, durable, protective coatings [67]. These coatings, reported by Appleget and Hodge, were highly tailorable due to the different components of the structure, so they had a wide range of potential applications depending on what coating quality was required. For example, specifically optimising the layer thickness of the crystalline and amorphous components led to an improvement in transparency. Generally, polycrystalline transparent ceramics have required high processing temperatures and maintenance of their homogeneity and optically isotropic crystal structure, making them sensitive and prone to degradation or porosity changes, increasing the light scattering and reducing transparency [68]. Transparent polycrystalline Y_2_O_3_ coatings combined with YSZ substrates have been shown to give improved strength and durability as compared to other ceramics. Coatings that were transparent in the UV and visible regions were deposited by Estropiev et al. by polymer salt synthesis [69]. The coatings were prepared by dip coating followed by a short drying step at 50 °C and then an annealing step at 550 °C. They comprised MgO-ZnO ceramic and had high durability and thermal stability. The highest transparency in the UV region was found for coatings with a relatively small MgO content (<20%) compared to the ZnO [69].

Photocatalytically active, transparent TiO_2_ coatings have been shown to degrade organic matter, making them effective self-cleaning coatings [40,70]. These coatings were deposited on a SiO_2_ buffer layer on polymer substrates and yielded a higher photocatalytic activity than the standard control. The inclusion of a buffer layer improved the compatibility between the photoactive coating and the polymeric substrate, and the separation of the TiO_2_ layer from the substrate had a positive effect as the intermediate SiO_2_; then, it behaved as a protective coating itself to the substrate against photocatalytic degradation [70]. These functionalised surfaces were applied in fabrics, furnishing materials, window glasses, roof tiles, car mirrors, and solar panels. The method described by Watté et al. involved dip coating of the SiO_2_ buffer layer followed by a heat treatment at 120–200 °C for one hour. The TiO_2_ layer was deposited by dip coating the already buffer-coated substrate with a nanoparticle suspension followed by a further heat treatment at 120–200 °C for one hour. The photocatalytic activity of the resultant films was analysed by the degradation of methylene blue (MB) dye and showed a significant improvement in the rate of photocatalytic activity over the control. The deposited coatings were also transparent; although they appeared yellow–brown when freshly prepared; after UV treatment to break down the residual organic material left by the deposition process, they became transparent, highlighting the photocatalytic ability [70]. Self-cleaning TiO_2_ coatings have also been deposited by sol–gels, as described by Adak et al., who followed the sol–gel dip coating with a nitrogen plasma treatment to improve the photocatalytic activity [40]. The plasma treatment improved the durability of the TiO_2_ layers, making them even more exciting candidates as protective coatings for photovoltaics. Regardless of the dip coating speed, the coatings were shown to have a higher transmission than bare glass and an extremely low water contact angle. Short nitrogen plasma treatments marginally improved the transmission, but this effect was lost as the plasma time increased beyond two and a half minutes. This was confirmed by refractive index changes, showing a reduction in refractive index compared to untreated porous TiO_2_ for plasma treatment times of up to two and a half minutes but an increase in refractive index beyond that. This was proposed to be due to the initial removal of adsorbed species by the nitrogen plasma, but as the treatment time increased, etching processes dominated, which increased the pore size, roughened the surface further and increased the refractive index [40]. Typically, self-cleaning coatings have required hydrophobicity to cause water to roll off the surface, removing the impurities. The TiO_2_ coatings also had a self-cleaning effect, but it was hydrophilicity based, inducing the water to permeate between the surface and the contaminants, causing the contaminants then to then wash off, as they were no longer adhered to the surface. Additionally, the TiO_2_ was photocatalytically active, which was demonstrated by the increased rate of MB dye degradation for the nitrogen plasma-treated coatings [40]. These coatings looked incredibly promising as barrier coatings for photovoltaics due to their high transparency, photocatalytic activity and self-cleaning nature and deserved serious consideration for future applications. Nitrogen plasma treatments have been used to good effect with other coatings, such as SiC deposited on glass–ceramic substrates by PECVD, which gave an improved chemical resistance after treatment with a nitrogen plasma and annealing at 400 °C. The annealing step was found to densify the coating, and the nitrogen plasma had a pore sealing effect, both of which improved the barrier performance of the coating on immersion in an alkaline solution [71].

**Figure 6:**
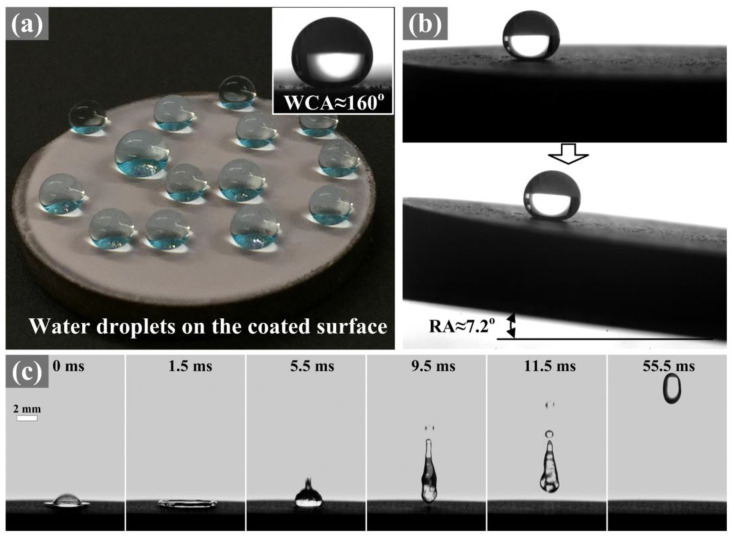
Wetting behaviour of the Yb(NO_3_)_3_ coating: (**a**) water droplets on the coated surface and WCA measurement; (**b**) RA measurement; (**c**) complete water droplet rebound after impact on the coating. Reprinted from [29], with permission from Elsevier.

Although ceramic coatings are typically hydrophilic due to their surface polarity, superhydrophobic ceramic coatings have been created for self-cleaning purposes. These coatings were deposited by injecting Yb(NO_3_)_3_ into plasma spray to form highly crystalline Yb_2_O_3_ surfaces with hierarchical surface roughness [29,72]. This roughness gave the surface superhydrophobicity: a water contact angle of ~160° and a low roll-off angle of ~7°, as shown in Figure 6. Exposure to a high temperature (>200 °C) allowed the surface to switch to from the Cassie–Baxter state to the Wenzel state, making it hydrophilic, although the coating was relatively stable at high temperatures and it took five hours to initiate the switch. This change was reverted by exposure to a vacuum, which restored the hydrophobicity of the surface.

The surface states were explained by the initial adsorption of hydrocarbons on the surface and trapped air in the surface roughness contributing to the Cassie–Baxter state of the surface. The adsorbed hydrocarbons were lost on extended heat treatment, allowing the water to permeate into the surface roughness and displace the air, resulting in a Wenzel state. Exposing the surface to a vacuum then allowed the readsorption of hydrocarbon species on the surface, restoring the superhydrophobicity [29]. This kind of reversible surface property combined with the durable nature of ceramics made it a very promising candidate as a barrier coating for environments where water exposure was likely and the temperature was likely to remain in the ambient range. Other self-cleaning hydrophobic and oleophobic coatings were developed using Ti_3_C_2_T_x_ MXene nanosheets. As well as demonstrating self-cleaning properties, these coatings showed a self-healing nature [73]. The MXene nanosheets were dip coated alternately with PDDA and an aqueous Ti_3_C_2_T_x_ MXene nanosheet suspension. This resulted in coatings formed from multiples of bilayers, and although they were transparent, there was a loss in light transmittance with each bilayer addition, resulting in approximately 50% transmittance for eight bilayers. When combined with a solid omniphobic slippery (SOPS) top layer, the coating showed a high level of omniphobicity, leaving a residue and streak-free surface after exposure to organic contaminants. Moreover, the coating appeared to be self-healing, restoring its omniphobicity on exposure to light after plasma oxygen etching. The plasma etching was designed to simulate surface oxidation on a more rapid scale than gradual exposure and showed an increase in surface energy and a subsequent loss in self-cleaning ability. Illumination under 1.5 sun was sufficient to restore the self-cleaning nature, as the elevated temperature allowed chain reorganisation. This was demonstrated to be repeatable with little loss in the omniphobicity [73]. This type of behaviour made these coatings highly promising as protective coatings for solar cells, although the inclusion of the fluoropolymer SOPS layer is a significant drawback due to their environmental impact of fluoropolymers and their corresponding increased regulations.

Wu et al. have reported another ceramic-based sol–gel deposited anti-icing coating with potential application as a protective coating for solar cells. This coating had a high transparency with ~98% light transmittance, had excellent substrate adhesion and mechanical robustness as well as self-cleaning and anti-icing capabilities [74]. The coating was formed by spray coating a sol of SiO_2_ nanoparticles functionalised with 1H,1H,2H,2H-perfluorooctyltriethoxysilane (PFOTES) to create the superhydrophobic surface. This functionalisation has also been applied to glass–ceramic coatings to impart hydrophobicity [75]. Different water contact angles were obtained depending on the length of time the solution was stirred for prior to coating; the longer time spent stirring gave a lower surface roughness due to better dispersion of the nanoparticles and fewer pores. The stirring time and resultant roughness also affected the transparency: the best transparency was obtained for samples which had had the longest stirring time. This meant a balance had to be struck between optimal transparency and water contact angle, although the ideal balance could change depending on the application [74]. Another drawback of this coating was the inclusion of fluoro-containing chemicals which were subject to increasing regulations around their use due to their long-lasting environmental impact. A mechanically, robust transparent ceramic composite coating could potentially be obtained by omission of the fluoro-functionalisation of the SiO_2_ nanoparticles, although it would lack the hydrophobic element.

TiO_2_ was used to functionalise polydimethylsiloxane (PDMS) nanoparticles to give a superhydrophobic transparent coating with anti-icing and self-cleaning properties [76]. They were formed by spray coating a TiO_2_ dispersion onto heat-treated PDMS, which was followed by a further heat treatment at 400 °C to give an enhanced surface roughness and a water contact angle of >150°. The coating was only hydrophobic (with a water contact angle of ~125°) without the secondary heat treatment. The heat-treated coating was responsive to UV exposure: the water contact angle dropped to ~130° after one hour UV exposure and then recovered after dark storage for 24 h. This was due to the photoactivity of the TiO_2_ causing the photogeneration of holes, which could react with surface oxygen to leave oxygen vacancies increasing the surface energy and reducing the contact angle. After removal of the UV source, the surface then adsorbed oxygen from the air and recovered the superhydrophobicity. The unfunctionalised PDMS coating had a transparency of 81%, which dropped to 76% after TiO_2_ functionalisation. It was reported that these coatings were also chemically stable, being able to withstand immersion in acid and alkaline solutions with no visible damage or significant change in hydrophobicity. They were also mechanically robust, withstanding water flow and sand impact without damage to the coating, as well as having a high adhesion to the surface [76]. Overall, these appeared to be promising coatings for photovoltaic applications. The transparency and requirement for heat treating was not ideal, but the durability, self-cleaning and water repellency could be very valuable qualities. The sand and water flow testing were very appropriate for solar cell applications, testing the samples in a “real-life” scenario. Optimisation to improve the transparency would be beneficial or else sacrificing some of the roughness and water repellency to improve the transparency. A slightly reduced contact angle of ~130° would still be water repellent but could recover a small amount of transparency, which would be very important to photovoltaic applications.

Another nanoparticle-functionalised transparent, superhydrophobic coating was reported by Polizos et al. [77]. This was deposited by spray coating SiO_2_ nanoparticles (both hydrocarbon- and fluoro-functionalised) dispersed in a ceramic–polymer matrix (Cerakote). The deposited coatings were rinsed and then dried at 65 °C prior to testing. The hydrophobicity was dependent on the proportion of polymer binder to nanoparticles; decreasing the amount of polymer binder increased the water contact angle, reaching a maximum of >140° for a binder to nanoparticle ratio of 2:1. This could be further improved to >160° by rinsing of the surface with solvent to remove excess binder and further expose the surface nanoparticles. However, decreasing the amount of binder too much resulted in a loss of coating durability, so the ratio needed to be balanced for the specific requirement. The durability was also affected by the curing time, and a 30–60 min cure gave the toughest samples. The percentage transmittance of the coatings was evaluated, and the rinsed samples showed an improvement over the uncoated glass and unrinsed samples [77]. This type of ceramic–polymer matrix which included hydrophobic nanoparticles was very applicable to photovoltaic applications due to the high transparency and superhydrophobicity. The low temperature required for the curing step was also appealing in terms of scaling up this coating to production levels and application to a variety of substrates. Further work evaluating whether comparable coatings could be fabricated with only the hydrocarbon-functionalised SiO_2_, excluding the fluoro-functionalisation, would be of interest. Modified SiO_2_ nanoparticles were dispersed in other polymer matrices, such as polysiloxane, as reported by Li et al. who deposited coatings formed from homogeneously dispersed epoxy-modified SiO_2_ in polysiloxane [78]. These coatings were shown to have an extremely high hardness and resistance to scratching as compared to the pure polymer coating, with a peak in scratch resistance found for coatings with 0.75 wt% SiO_2_ nanoparticle incorporation. This iteration also had a reduced penetration depth during nanoindentation testing and the best abrasion resistance of all the tested coatings, supporting the 0.75 wt% coating as the optimal choice in terms of coating durability. These coatings also had excellent transparency, with up to 2 wt% modified SiO_2_ incorporation showing a transparency over 90% despite the high thicknesses of the coatings at ~200 µm [78]. The transparency and durability of these coatings made them excellent candidates for photovoltaic applications looking for long-term stability. However, the brush-coating method of coating deposition was not ideal for large-scale coating manufacture, so other methods of fabrication should be evaluated to optimise for large-scale production.

Coatings and surface functionalisations have been used to impart UV protection to photovoltaic cells. This is beneficial due to the damage incurred by photovoltaic cells by UV degradation of the encapsulants, reducing the lifetime of the cell. Ideally, the coating would significantly reduce the amount of UV light transmitted to the underlying device without reducing the amount of visible light. Two such coatings were investigated by Johannson et al. [79]. The ZnO and TiO_2_ coatings were deposited by spray pyrolysis and were shown to reduce transmittance in the UV region by ~85% and ~75%, respectively; however, there was also a 12.3% and 21.8% respective reduction in transmitted convertible light. The coatings were typically <100 nm, and they were reported to have several voids and defects, and most iterations had a low RMS roughness of <10 nm. Although the ZnO coatings had a higher UV absorption than the TiO_2,_ the TiO_2_ had a slightly higher reflectivity in the UV region than the ZnO. This was proposed to be due to the refractive index mismatch between the TiO_2_ and the soda lime float glass substrate [79]. These functionalisation methods could be highly beneficial to photovoltaic cells despite the reduction in transmission of convertible energy due to the potential increase in cell longevity.

#### 2.2.2. Glasses

Surface coatings and treatments on glass surfaces can be of significant benefit to photovoltaic cells due to their potential to impart self-cleaning and anti-icing properties. One of the major difficulties to overcome in their widespread application and use was the balance between increasing the roughness to improve the functionality and maintaining the transparency of the glass. Another problem was the durability of the coatings as when applied to protect photovoltaics, they must withstand mechanical and chemical wear. Additionally, they must be defect free to provide full protection as well as chemically stable and benign to avoid the release of damaging pollutants into the environment in the event of damage or degradation [11].

Silicate glass networks consisting of SiOC structures have been reported to show improvements over standard glass coatings. Stabler et al. reported them as having an improved Young’s modulus, hardness and glass transition temperature [51]. The inclusion of tetravalent carbon sites was attributed to the increase in Young’s modulus of SiOC glasses when compared to vitreous SiO_2_ due to the higher connectivity of the glass network [80]. SiOC-based coatings were shown to range from insulating behaviour to potentially acting as a semi-conductor material. This was reported by Cordelair and Greil and was mostly attributed to the carbon content and degree of ordering in the segregated carbon phase [81]. The semi-conducting effect could possibly be caused by p-type carrier conduction in the structure, which is why it was so dependent on the amount of carbon in the material [82]. Furthermore, chemical durability and etching resistance have been reported by Sorarù et al., which performed better than the fused SiO_2_ or soda–lime glass comparisons. Again, this was attributed to the carbon content and the phase separation, as the tetrahedral Si-O bonds were preferentially attacked, and SiOC structures with a greater carbon content were more resistant to chemical attack or etching [83]. Bar coating has been used to deposit a functional SiO_2_-based film on glass for photovoltaic modules from a SiO_2_-based suspension [84]. This coating was reported by Yoo et al. as having an excellent hardness, adhesion, high transparency and desirable contact angle after a 300 °C two-minute cure. The coated sample had an excellent self-cleaning effect which was attributed to the low contact angle. Additionally, this coating had 99.8% transparency as compared to untreated glass, so it could be a quick, low-cost, and simple way to functionalise glass coatings for photovoltaic applications [84].

Dimethyldichlorosilane (DDS)-functionalised SiO_2_ nanoparticles were used to develop self-cleaning coatings with a high durability [85]. The substrates were functionalised with an adhesive before the nanoparticle suspension was sprayed on to deposit the coating with no requirement for a curing stage; it was simply dried for five minutes in air. These coatings were designed for oil/water separation applications and so had a very high water contact angle of ~160° and a highly oleophilic characteristic, which was demonstrated by a coated sponge which absorbed oil from an oil/water mix with no significant water uptake. The coatings were mechanically robust and had some ability to withstand abrasion with sandpaper. All of the samples maintained hydrophobicity, plateauing at ~120° after 200 abrasion cycles, but it took a sample coated six times to withstand 300 abrasions cycles without losing the superhydrophobicity. The coatings were deposited on glass and demonstrated a high transparency for one and two coating cycles, but this started to decrease with further coating cycles [85]. These coatings would be potentially applicable for photovoltaic applications and could be used to functionalise a glass encapsulant. The drawbacks of this type of coating were the halogenation, as the SiO_2_ nanoparticles required chlorine functionalisation, and the loss of transparency found for the more robust coatings formed by repeated coating cycles. Similarly, a two-layer coating system was prepared by Wang et al., where instead of a sprayed adhesive, an initial polymer layer of γ-(2,3-poxypropoxy) propyltrimethoxysilane in polyethylene glycol was dip coated onto a substrate, which was followed by immersion in a sol of long-chain and hydrophobic functionalised SiO_2_ to form the top layer [86]. This coating had a high light transmission with both the hydrophobic SiO_2_ only and the hydrophobic SiO_2_ plus long-chain SiO_2_ combination. The anti-reflective quality of the coating was demonstrated by the increased transmission as compared to uncoated glass. The combination coating had a hydrophobic surface with a water contact angle of >140° when a <12 mL long-chain SiO_2_ sol was included in the reaction mixture. The resulting self-cleaning ability was demonstrated by using water droplets to remove various contaminants from coated glass, showing a significant improvement over uncoated glass. Although the long-chain functionalised SiO_2_ was slightly detrimental to the hydrophobicity, it resulted in an improvement in durability, and it increased the toughness of the coating with an optimal balance found for 8.3 mL long-chain SiO_2_ sol. These coatings also had a high degree of chemical resistance, showing no damage on exposure to acid or alkaline solutions [86].

SiO_2_ nanoparticles were functionalised with hexamethyldisilazane (HMDS) to make them hydrophobic, anti-reflective coatings for photovoltaics [87]. The functionalised nanoparticles were dispersed in an organosilica binder and deposited by dip coating. The coating had a tailorable refractive index which changed in relation to the proportion of organosilica binder. A low refractive index was achieved by using a low amount of binder due to the high porosity and loose stacking of the nanoparticles. The coating had a very high transmittance, peaking at 99.9% with a binder proportion of 30–40 wt%. Increasing the binder proportion also increased the hardness of the coating, making it more durable. This increased linearly up to about 80 wt%. The durability was tested by rubbing the surface with felt up to 500 times with the coating showing no visible damage, no loss in transparency and maintenance of the hydrophobicity (although the superhydrophobic effect was lost, the water contact angle dropped from >160° to 135°). The self-cleaning ability of the coating was demonstrated by rinsing the superhydrophobic surface with water droplets to remove dust and contaminants. The coating was shown to outperform conventional SiO_2_ coatings when compared in a real-life outdoor test for three months with only a slight loss in transparency [87]. These types of functionalisations were very promising for photovoltaic applications due to their hardness, transparency and superhydrophobicity without having to use fluorine.

Glasses can be functionalised to give them more desirable properties, such as colour changes, hydrophobicity, surface texturing, anti-reflectiveness etc. Photoluminescent glasses have been achieved by Al-Qahtani et al. [88] by coating with lanthanide-doped strontium aluminium oxide (LSAO) in a polystyrene (PS) nanocomposite by ultrasonic-assisted solution blending. As well as photoluminescence, these coatings were hydrophobic and showed improved scratch resistance over uncoated glass. The coatings with the highest ratios of LSAO in the blend showed the greatest scratch resistance as well as the highest tensile strength and highest water contact angle. At low contents of the LSAO, the coated glass displayed fluorescent photochromic emission [88]. There was potential to use this coating functionalisation on protective coatings for photovoltaic devices to improve their durability and hydrophobic behaviour without negatively affecting the transparency of the glass.

#### 2.2.3. Glass–Ceramics

Glass–ceramics have been historically used as coatings for wall claddings to provide colour, gloss, chemical resistance and UV resistance to the structure underneath due to their durability, longevity and cost effectiveness [2]. Transparent glass–ceramics can also be used in photonics due to the presence of the crystalline structure [18]. The mechanical properties of glass–ceramics can be strictly controlled, making them suitable for many applications. Typically, the mechanical strength of a glass–ceramic was higher than that of the parent glass material; due to the nanocrystalline phase, the composite should have higher fracture toughness and flexural strength [18]. This increased the appeal of glass–ceramic coatings specifically as protective coatings for devices which also require a high level of optical transparency, such as photonics or as barrier coatings for photovoltaic devices. The glass–ceramic coatings reported by Gülen et al. showed a high stability to UV exposure in terms of gloss and colour maintenance but showed a change in water contact angle over hundreds of hours UV light exposure. The samples with the lowest wt% TiO_2_ had the most stable contact angle, so they would be the candidates that would be best applied as protective coatings in photovoltaic applications. Fortunately, the samples with the lowest wt% TiO_2_ had the highest transparency.

Yan et al. demonstrated that chemically bonded phosphate ceramics could be reinforced with glass flake to give a glass–ceramic structure with an improved corrosion resistance [89]. As with the addition of TiO_2_ nanoparticles to the Al_2_O_3_/ZnO phosphate ceramic described by Liu et al. [66], the addition of MgO and glass flake gave a denser, more compact coating with an enhanced protective performance. This was because the MgO and glass flake increased the reaction temperature which allowed a higher degree of crosslinking. A new bonding phase introduced by MgO and the physical presence of glass flake created a more tortuous path for permeant molecules, improving the anti-corrosion behaviour of the coating. EIS was used to demonstrate the enhancement in resistance over uncoated steel substrates and the ceramic alone, with an increase in glass flake concentration giving an increased impedance up to a glass flake concentration of 15 wt% [89].

A SiO_2_-Na_2_O-B_2_O_3_-Al_2_O_3_-CaO glass–ceramic system was stabilised with WC nanoparticles to give a highly abrasion resistant coating [43]. The coatings were deposited by wet spraying and crystallised at over 700 °C on iron substrates. A small amount (2.5 wt%) of WC incorporated in the structure had the best effect on the coating durability [43]. Y_2_SiO_5_/Y_2_O_3_-Al_2_O_3_-SiO_2_ glass–ceramic coatings reinforced by β-Y_2_Si_2_O_7_ nanowires have been used as oxidation barriers, as described by Zhou et al. [90]. These coatings were deposited by a combination of pulse arc discharge deposition and hot dipping to create a tough, multiphase system. The inclusion of the nanowires in the coating reduced the amount of cracking and defects, and the dense glass–ceramic top layer acted as an oxygen diffusion barrier. The resulting coatings were able to withstand over 150 h at >1670 K with only marginal mass loss [90]. A dense glass–ceramic coating described by Chen et al. was shown to significantly improve the oxidation resistance of the underlying steel substrate at 650 °C. The coating was a spray-deposited milled glass comprising SiO_2_ (67.5%); Na_2_O (12.5%); Al_2_O_3_ (6%); K_2_O (1.5%); MgO (1.5%); CaO (7%); and BaO (1%), which was melted to form a glass ceramic coating, which impeded the diffusion of O^2−^ ions and allowed selective oxidation at the interface between the coating and the substrate to form a corrosion barrier [91]. Y_2_O_3_-Al_2_O_3_-SiO_2_ (YAS) glass–ceramics have been described by Deng et al. as protective barrier coatings used to prevent calcium magnesium aluminosilicate (CMAS) corrosion. It was found that increasing the Y_2_O_3_ concentration in the fabrication process increased the nucleation rate, resulting in an overall smaller grain size. This led to an increased surface area, accelerating the corrosion reaction when exposed to CMAS melt [92]. Feng et al. described a glass–ceramic coating comprising SiO_2_-Al_2_O_3_-ZrO_2_-Ba(Sr, Ca)O doped with Cr_2_O_3_ [93]. This addition of the Cr_2_O_3_ was found to improve the wear resistance and lower the coefficient of friction. Without the addition of Cr_2_O_3_, when annealed at >1000 °C, BaSi_2_O_5_ crystallites were formed, but with Cr_2_O_3_ included in the fabrication process, the major crystal products were BaCrO_4_ and BaCr_2_O_4_, which promoted the formation of a lubricating layer and reduced the rate of wear damage [93].

α-cordierite glass–ceramic coatings were deposited on porous BN/Si_2_N_2_O ceramic substrates. Theses coatings were reported to give improved thermal stability and chemical resistance. The properties of the glass–ceramic coating were very dependent on the processing temperature during the coating deposition. The coating’s phase composition, microstructure, mechanical properties and dielectric properties were all affected by the processing temperature of the glass–ceramic deposition [94]. Melt quenching was used to fabricate glass–ceramic coatings comprising SrO-Bi_2_O_3_-B_2_O_3_, which were moderately transparent. Due to the photocatalytic activity, these coatings were recommended for self-cleaning applications on glass substrates [95]. Other glass–ceramics which showed transparency and photocatalytic activity have been reported previously, such as 70B_2_O_3_–29Bi_2_O_3_–1Dy_2_O_3_)–x(BaO–TiO_2_) [96], 20WO_3_–50ZnO–30B_2_O_3_ [97], (42-x)P_2_O_5_–8MgO–50ZnO–xTiO_2_ [98], 70TiO_2_–30P_2_O_5_ [99] and SnO-doped CaO–B_2_O_3_–Bi_2_O_3_–Al_2_O_3_–TiO_2_ [100]. The SrO-Bi_2_O_3_-B_2_O_3_ coating reported by Singh et al. showed a good photoreduction in resazurin-based ink confirming the self-cleaning ability and a 60–80% transparency with a yellowish tinge as compared to uncoated glass. Due to this lower than ideal transparency, discoloration, and high processing temperatures (1000 °C), this coating would not be ideal as a protective coating for photovoltaic applications.

The sol–gel dip-coated 80SiO_2_-20LaF_3_ doped with Nd^3+^ had a tuneable refractive index depending on the temperature during the post-deposition heat treatment step and the resultant SiO_2_ structure and degree of LaF_3_ crystallisation [54]. These oxyfluoride glass–ceramic coatings were optically efficient and highly transparent [37,38]. Their tuneable nature in response to heat treatment was of potential use in photonic applications where a specific refractive index would be required, such as in fibre amplifiers or tuneable lasers.

One method of improving the fracture toughness of glass ceramics was to design surface compression. Surface compression prevented crack initiation, as a higher energy was required to propagate defects. Creating surface compression by tempering or ion exchange has been a commonly used method to strengthen glasses, but these were not as effective on ceramic coatings. The same principle has been applied, however, by exploiting a CTE mismatch between two bonded materials by cooling them from a high temperature, as the material with the higher CTE shrank most and caused compressive stress in the other material. Du Merac et al. investigated this by coating a transparent ceramic YSZ with thin, low CTE Y_2_O_3_ coatings. The Y_2_O_3_ coatings were deposited by electron-beam PVD and were dense and highly transparent. They showed little significant difference to the uncoated YSZ other than a slightly higher light transmittance in some samples, which was attributed to reduced reflections because of the lower refractive index of Y_2_O_3_. This could be optimised to improve transmittance further by optimisation of the coating thickness. A significant reduction in crack propagation was found after heat/cool treatment of the Y_2_O_3_-coated YSZ as compared to heat/cool-treated YSZ only [68]. An oxidation barrier was formed from a Y_2_Ti_2_O_7_ and SiO_2_-based glass–ceramic coating with Y_2_Ti_2_O_7_ as the principal crystalline phase. The coating deposited by melt casting unfortunately required high manufacturing temperatures of >1000 °C but withstood an oxidising environment at 600 °C for 120 h without showing any significant degradation, proving its effectiveness as an oxidation barrier [101].

Glass–ceramic coatings formed from recycled industrial waste were applied as durable, protective thermal barriers. The composition was variable, depending on the feedstock, with general wt% proportions of SiO_2_ (35–60%), Al_2_O_3_ (2–15%), Fe_2_O_3_ (1–26%), CaO (9–25%), MgO (1–20%), and R_2_O (0–12%) [102]. The coatings were found to have an extremely high thermal resistance, which was attributed to the closed porous nature of the coatings. The coating was stable over 30 cycles of 23–700 °C, demonstrating the thermal barrier characteristic. Additionally, the coatings were well adhered to the substrate, had a hardness of >400 MPa for all compositions and a reported 54% improvement in abrasion resistance. The chemical stability was also tested and showed a high resistance in boiling water, acid and alkaline solutions. The durability and stability improvements were attributed to the mechanical strength of the crystalline phase [102]. However, despite the good performance of the coatings as protective barriers, high processing temperatures of >800 °C were required, which was unsuitable for many applications and substrates. Additionally, no mention was made of the transparency or opacity of the coatings.

Li_2_O-Al_2_O_3_-SiO_2_ glass–ceramics have been shown to have a very low CTE, which made them excellent for thermal barrier applications. The addition of TiO_2_ or ZrO_2_ to this glass–ceramic as nucleation agents resulted in a final transparent coating due to the small crystal size reducing scattering. The difficulty resided in the sintering process: to maintain the small crystal size, methods such as spark plasma sintering must be used, which are difficult to apply on an industrial scale [7]. CaO-B_2_O_3_-SiO_2_ (CBS) glass–ceramic coatings had a relatively low dielectric constant and high thermal expansion but could be modified by the incorporation of Al_2_O_3_ [31,32]. The inclusion of Al_2_O_3_ over a 50–70 wt% range led to a density increase as well as a slight increase in porosity, although this levelled out after heat treatment caused a densification of all samples. The dielectric constant peaked at an Al_2_O_3_ content of 65 wt%, showing a 60% increase over the 1:1 mix ratio [31]. A coating with a tailorable dielectric constant and porosity could be useful as an electrical barrier coating, but no mention was made of transparency, and the thickness was quoted at 660 µm, which made these coatings less practical for photovoltaic applications.

A non-exhaustive summary of the applications of ceramic, glass and glass–ceramic coatings presented in Table 3 and separated into their broad classifications.

The coatings designed specifically for use in conjunction with photovoltaics are highlighted in Table 4. As not every data set reported the transparency and reflectivity in the same way, the values at 400 nm and 600 nm were selected, as these were common to most articles. The self-cleaning capability was typically measured either in MB dye degradation or by visually displaying dust or other contaminants being removed from the surface by water. Unless otherwise specified, the contact angle refers to the water measurement. The preferred options (indicated by *) were those which had either a high transparency or low reflectivity, or both, at a low coating thickness. Of these, the coatings which also had a degree of hydrophobicity were deemed the most favourable for solar cell protection.

## 3. Discussion

The SiO_2_-based coatings discussed generally showed the best potential due to their intrinsic hydrophobicity and barrier properties. One of the most promising coatings reported in the recent literature was the ceramic SiO_2_-based coatings described by Channa et al. [64]. These coatings were highly impermeable to oxygen and water and were transparent, making them ideal as barrier coatings for solar cells. In the research described, they had been applied to solar cells and were specifically designed for this application. The low-temperature curing stage made these coatings appropriate for sensitive substrates; however, flexibility was not assessed. A wider range of applications would be available if these coatings were developed to accommodate bending and flexing. However, this capability was demonstrated by the SiO_2_-based coating described by Kim et al. [63], which showed significant potential as a barrier coating for organic solar cells. These anti-reflective coatings comprised alternating thin, ALD fabricated Al_2_O_3_/SiO_2_ layers and were subjected to harsh environment testing to stress the multilayered coating as much as possible to fully assess the capability. The coating withstood exposure to high temperature/high humidity environments and bend testing with no loss in performance. Aside from being a protective barrier, the coating described was an enhancement to the solar cell, showing a decrease in reflectivity over an uncoated cell. Although this coating did not have the highest transparency or lowest reflectivity of the assessed coatings, it stood out because of the demonstrated flexibility. This was the best assessed coating and set the standard for other coatings; aside from protecting the underlying cell from environmental damage, they improved the cell performance. Similarly, alternating layers of TiO_2_/SiO_2_ showed significant potential as transparent protective coatings for solar cells. These thin coatings had a very high transparency and low thickness [58,59,142], making them extremely promising. There was no mention, however, of hydrophobicity or self-cleaning effects, so this coating could benefit from a combination with a top layer to impart these qualities and further research to include intrinsic hydrophobicity in the top layer or further exploit the photocatalytic activity of the TiO_2_ layer.

Other SiO_2_ layers showed significant potential as barrier coatings for solar cells. Hydrophobic SiO_2_ was reported by Wang et al. [86] by dip coating. These coating were highly transparent, resistant to chemical attack and had a water contact angle of >150°. The thickness was not specified, but this coating could be a useful addition to other coating systems as a transparent hydrophobic top layer. In the reported work, it was deposited on a polymer base layer, but it could potentially be used as the SiO_2_ component of a TiO_2_/SiO_2_ or Al_2_O_3_/SiO_2_ multilayer coating system which otherwise lacked hydrophobicity. Dip-coated double-layer SiO_2_ coatings that showed a self-cleaning effect demonstrated by contaminant removal combined with high transparency were reported by Liu et al. [139].

Another highly effective SiO_2_-based coating was deposited by Kócs et al., although this was a single-layer coating [152]. The transparency of this coating was high and the reflectivity was low, but no mention was made of a self-cleaning effect or hydrophobicity. If this functionality was required, it could be combined with another coating type as part of a multilayer system or functionalised to impart hydrophobicity.

SiO_2_ nanoparticles were used to give self-cleaning coatings, as reported by Chen et al., who functionalised SiO_2_ nanoparticles with DDS to give an extremely superhydrophobic coating [85]. The drawback to this highly effective coating was the use of halogens in the functionalisation, but this could potentially be outweighed by the ease of the deposition, as it required no heat-curing step after the simple spray coating. The coatings had a high durability, showing maintenance of hydrophobicity after abrasion with sandpaper, but this could in part be due to the other drawback of this method: the pretreatment of the substrate with an adhesive. Without this extra step, it was unclear how the coating would behave in response to physical damage. A more appealing high durability alternative was reported by Chi et al., who functionalised SiO_2_ nanoparticles with HMDS to give an extremely hydrophobic and very highly transparent coating [87]. The durability of this coating was assessed by physically rubbing the surface, demonstrating the hardness. The combination of hydrophobicity, durability and transparency made this coating very promising either as a standalone or in conjunction with other layers.

Al_2_O_3_ multilayered coatings were discussed by Reuna et al. and shown to have an extremely high transparency and low reflectivity [145]. These coatings were deposited by sputtering and were reported to have nano-structuring, although no mention was made of hydrophobicity. The combination of these layers with other thin coatings could give an enhanced coating with a greater range of functionalities.

Self-cleaning, photocatalytic TiO_2_ coatings were also commonly used, whether as stand-alone coatings, composites or functionalisations. PDMS functionalised with TiO_2_ was very effective at forming protective self-cleaning and anti-icing coatings [76]. They were reported to be superhydrophobic, chemically resistant and mechanically tough, which made these coatings valuable candidates as barrier coatings for solar cells. However, they were only moderately transparent and required heat treatment at 400 °C to achieve their full functionality. Without the 400 °C heat treatment, the coatings had some degree of hydrophobicity, and it is possible that with further optimisation or thickness reduction, the transparency could be improved. Photocatalytically active, transparent TiO_2_ coatings were fabricated by dip coating a suspension of nanoparticles, which was followed by a heat treatment to 120 °C [70]. This was used to functionalise a SiO_2_ substrate but could also be deposited on other substrates to impart the photocatalytic cleaning ability. An existing protective barrier coating could be treated with this nanoparticle layer to give additional functionality. This was also demonstrated by sol–gel-deposited TiO_2_ described by Adak et al. [40], who further treated the TiO_2_ layer with a nitrogen plasma. The Innovative nitrogen plasma treatment of the TiO_2_ improved the photocatalytic activity, the durability, and resulted in higher transmission of incoming light by decreasing the reflectivity. Both the use of sol–gels to deposit the coating and the nitrogen plasma treatment were good techniques, as they were cheap and versatile; sol–gel nanoparticle depositions could be adapted to multiple wet coating methods, and any surface architecture could be treated with a short nitrogen plasma due to the nature of the vacuum technique.

Crystalline Yb_2_O_3_ coatings were used to fabricate superhydrophobic coatings with hierarchical surface roughness [29]. In the ambient temperature range, these coatings were stable and could be applied as a functional layer on a glass encapsulant or existing barrier coatings to impart self-cleaning capability. MgO-ZnO coatings were very versatile, and they were able to be deposited by sol–gel wet coating to give mechanically stable, transparent coatings. These coatings were thermally stable in their final form but required an annealing step of >500 °C after the deposition [69]. This made them inappropriate for temperature-sensitive substrates and flexible solar cells, but they could be applied to thermally stable substrates if required.

Epoxy–ceramic composites were another promising candidate for cell protection. They showed a good degree of flexibility, high transparency, anti-fouling capability, mechanical durability and UV resistance, so the potential was significant [60]. Although the main discussion by Chen et al. was regardingZrO_2_–epoxy composites, it would be worth investigating the properties of other ceramics in this configuration. For example, there could potentially be a benefit to including TiO_2_ to impart some photocatalytic activity for self-cleaning purposes, or including SiO_2_ to increase the hydrophobicity, or combinations of these ceramics to obtain the best possible coating. As long as the key characteristics, such as transparency, flexibility and impermeability could be maintained, there would be almost endless possibilities to tailor the coating to the application.

## 4. Conclusions

There were many methods of coating deposition which were used to fabricate barrier coatings but the best methods were those which are widely applicable, scalable, and low cost. Many methods required high temperatures, over 500 °C, either in the deposition phase or as a post-deposition curing or annealing step. Whilst this may be suitable for silicon cells or traditional substrates, these temperatures were less suitable for plastic substrates, which have been increasingly used for flexible solar cells. Aside from substrate compatibility, lower-temperature deposition methods were more practical in terms of cost and energy consumption, which made them more desirable for scale up to production.

There have been several different coating types presented herein, with different attributes and applications. The properties which are required or desirable for a protective or enhancing coating for photovoltaic applications are as follows:

- High transparency;
- Low water permeation;
- Low oxygen permeation;
- Hydrophobic or superhydrophobic;
- Self-cleaning/anti-fouling activity;
- Anti-reflective/low reflectivity of useful incident light;
- Light downshift;
- Corrosion resistance;
- Chemical resistance;
- Wear resistance;
- Scratch resistance.

To achieve the highest level of solar cell protection it could be necessary to use multiple different coating types to combine their properties. As long as the transparency is maintained there are potentially many combinations which would be beneficial, such as the combination of a moisture barrier with a hydrophobic top layer. However, an increased number of layers leads to an increased number of potential interfacial reflections and optical losses, so layer selection must be judicious. The most promising and exciting of the reviewed coatings were those which were multilayered and SiO_2_ based, as many of those were highly transparent, durable, and adaptable to several deposition methods. The combination of two or more of the best methods could be a way to further exploit the coating qualities to give further improvement in cell protection.

## Figures and Tables

**Table 1:** Properties of ceramics, glasses and glass–ceramics [5].

Property	Ceramics	Glasses	Glass–Ceramics
Hardness	High	High	High
Elastic modulus	High	High	High
Ductility	Low	Low	Low
Wear resistance	High	High	High
Corrosion resistance	High	High	High
Chemical resistance	High	High	High
Weather resistance	High	High	High
Melting point	High	Medium–High	Medium–High
Working temperature	High	Medium–High	Medium–High
Thermal expansion	Low	Medium–Low	Medium–Low
Thermal conductivity	Medium–Low	Low	Medium–Low
Electrical insulation	High	High	High
Tensile strength	Low–Medium	Low	Low–Medium
Compressive strength	High	High	High
Transparency	Low	High	Low–High
Brittle	High	High	High
Impact strength	Low	Low	Low
Thermal shock resistance	Low	Low	Low

**Table 2:** Key features of common ceramic, glass and glass–ceramic deposition techniques.

Method	Key Features	References
Cold gas dynamic spraying (CGDS)	Low temperature process Low coating stress High deposition efficiency High coating density and purity Controlled phase and microstructure Controlled thickness Wide range of material and substrate compatibility Coating properties influenced by spray velocity, temperature, oxidation level and particle morphology Can require post-process heat treatment	[20,21]
Electrophoretic deposition	Controlled thickness Controlled quality and microstructure Complex, functionally graded coatings achievable Solution-based batch process Coating affected by process conditions: voltage, solution strength, etc.	[22]
Plasma spraying	Controlled thickness Controlled quality and microstructure by control of feedstock particle size Rough, highly temperature stable coatings Excellent mechanical performance achievable Hierarchical surface structuring achievable Requires high temperature curing/crystallisation stage	[23,24,25,26,27,28], [29,30,31,32]
Aerosol deposition	Fabricate dense films at room temperature High adhesion strength High deposition rate and wide thickness range Patternable via rendering or masking etc. without etching Can deposit in low vacuum to atmospheric pressure	[33]
Sol–gel	Low cost and low temperature processing Can produce good quality, crack-free coatings Extremely versatile and compatible with many other methods, such as dip coating or spin coating Can be used to form transparent glass–ceramics High temperatures can be required post-processing	[4,18,19,34,35,36,37,38,39,40,41]
Wet spray	Glass–ceramic colour and transparency easily controlled by altering the raw material’s chemical composition Dense, defect-free coatings A bond coat can be required to promote adhesion Can require a crystallisation step at high temperatures	[2,42,43]
Laser assisted CVD	Excellent structure and polymorph control Thickness control Rapid growth rate Dense, defect-free films Good substrate adhesion	[44]
Plasma assisted CVD	Low deposition temperatures make it suitable for temperature-sensitive substrates Dense/conformal coatings with good uniformity Good substrate adhesion Structural properties affected by the precursor ratio	[45]
Hot dip plating	Dense, smooth coatings Coatings are uniform and well-ordered with high temperature stability Thickness easily controlled by duration of reaction Requires high temperature oxidation	[46]
Laser cladding	Simple process Coatings have good mechanical and thermodynamic properties, high strength, wear resistance, etc.	[47]

**Table 3:** Summary of ceramic, glass and glass–ceramic coatings and their applications.

Application	Coating Type	Deposition Method	Refs.
Electrical/corrosion protection	Al_2_O_3_-TiO_2_	Plasma spray	[26,103,104]
	WC-Al_2_O_3_	Plasma spray	[27]
	CBS/Al_2_O_3_	Plasma spray	[31]
	CaO–B_2_O_3_–SiO_2_	Plasma spray	[32]
	Al_2_O_3_ Aluminium nitride	Aerosol	[33]
	Al_2_O_3_/SiO_2_	ALD	[63]
	Polymer ceramic SiO_2_	Blade coating	[64]
	γ-Al_2_O_3_ with epoxy resin topcoat	Electrolytic	[65]
	Al_2_O_3_/ZnO phosphate ceramic reinforced with TiO_2_ nanoparticles	Powder coating	[66]
	Glass flake nanoparticle-reinforced MgO phosphate ceramic coating	Brush coating	[89]
	SiOC Glass	Pyrolysis	[81]
	SiO_2_-Na_2_O-Al_2_O_3_-K_2_O-MgO-CaO-BaO	Melt quench/spray	[91]
	PRMMC Al_2_O_3_-Cu PRMMC Al_2_O_3_-Al PRMMC Al_2_O_3_-Ni PRMMC Al_2_O_3_-Ni-Zn	CGDS	[105,106,107,108,109] [110] [111,112]
	Mercapto-functionalised SiO_2_	Sol–gel	[113]
	Al_2_O_3_	Sol–gel	[114,115,116]
Wear resistance/scratch resistance	Li_2_O-SiO_2_	Crystallisation	[7]
	Cr_2_O_3_ TiO_x_ Cr_2_O_3_–TiO_x_–Al_2_O_3_ Ternary coating	Plasma spray	[24]
	ZrB_2_-SiC-TiSi_2_	Plasma spray	[25]
	Al_2_O_3_-TiO_2_	Plasma spray	[26]
	Al_2_O_3_-GdAlO_3_	Plasma spray	[28]
	SiO_2_, ZnO, Al doped ZnO, or Al_2_O_3_ coating on TiO_2_/Ag/TiO_2_ stacks for IR reflectivity	Sol–gel	[39]
	TiO_2_-doped ZrO_2_	Sol–gel	[41]
	SiO_2_-Na_2_O-B_2_O_3_-Al_2_O_3_-CaO with WC nanoparticles	Wet spray	[43]
	TiAlCN	Plasma-assisted CVD	[45]
	Al_2_O_3_-TiB_2_-TiC	Laser cladding	[47]
	Y/Sialon	Pulsed laser deposition	[57]
	Zirconium epoxy–ceramic	Droplet	[60]
	Polycrystalline Y_2_O_3_ on YSZ ceramic substrates	Electron-beam PVD	[68]
	PFOTES-functionalised SiO_2_ nanoparticles	Sol–gel	[74]
	TiO_2_ functionalised PDMS	Wet spray	[76]
	Epoxy modified SiO_2_ in polysiloxane	Brush coating	[78]
	DDS-functionalised SiO_2_ nanoparticles	Wet spray	[85]
	Hydrophobic and long-chain functionalised SiO_2_ on a polymer base layer	Dip coating	[86]
	HDMS-functionalised SiO_2_ nanoparticles in organosilica binder	Dip coating	[87]
	Y_2_O_3_-Al_2_O_3_-SiO_2_	Melt quench	[92]
	Cr_2_O_3_-doped SiO_2_-Al_2_O_3_-ZrO_2_-Ba(Sr, Ca)O	Melt quench	[93]
	Y_2_Ti_2_O_7_-SiO_2_	Melt casting	[101]
	SiO_2_-Al_2_O_3_-Fe_2_O_3_-CaO-MgO-R_2_O	Dip coating	[102]
	PRMMC Al_2_O_3_-Cu PRMMC Al_2_O_3_-Al PRMMC WC-Ni	CGDS	[107,117] [107,109,118] [119,120]
	Gradient TiO_x_-Al_2_O_3_	Thermal oxidation	[121]
	Transparent TiO_2_/Al_2_O_3_	Electron beam evaporation	[122]
	SiO_2_ nanoplates in cellulose	Sol–gel	[123]
Electrical conductivity	BaAl_11_O_17_ YBa_2_Cu_3_O_7_−δ	Laser-assisted CVD	[44]
	SiOC Glass	Pyrolysis	[81]
	PRMMC Al_2_O_3_-Cu	CGDS	[105]
Intermediate compatibility layer	PRMMC Al_2_O_3_-Al PRMMC Al_2_O_3_-Ni PRMMC Al_2_O_3_-Ni-Zn	CGDS	[124,125]
Photocatalyst	Porous TiO_2_	Aerosol	[33]
	TiO_2_ with nitrogen plasma treatment	Sol–gel	[40]
	TiO_2_-functionalised PDMS	Spray	[76]
	Porous TiO_2_	Sol–gel	[126]
	TiO_2_	CGDS	[127,128]
	GaN	CGDS	[129]
Biomedical	Rare earth-doped glass–ceramics	Sol–gel	[18]
	Ceramic HA	CGDS	[130]
	Al_2_O_3_	Sol–gel	[131]
	TiO_2_	Dip coating	[132]
	ZnO-PTFE composites	Sputtering	[133]
Solid lubricating coatings	TiAlCN	Plasma assisted CVD	[45]
	Cr_2_O_3_ doped SiO_2_-Al_2_O_3_-ZrO_2_-Ba(Sr, Ca)O	Melt quench	[93]
	PRMMC Al_2_O_3_-Cu-graphite PRMMC Al_2_O_3_-(Cu-5Sn)-Ag	CGDS	[134] [135]
Chemical/UV protection/IR protection	Glass ceramics composed of mainly TiO_2_, Na_2_O/K_2_O/Li_2_O, SiO_2_/ZrO_2_, and Fe_2_O_3_/Al_2_O_3_/B_2_O_3_	Wet spray	[2]
	SiO_2_, ZnO, Al doped ZnO, or Al_2_O_3_ coating on TiO_2_/Ag/TiO_2_ stacks for IR reflectivity	Sol–gel	[39]
	Zirconium epoxy–ceramic	Droplet coating	[60]
	TiO_2_ functionalised PDMS	Wet spray	[76]
	TiO_2_ functionalisation on glass ZnO functionalisation on glass	Spray pyrolysis	[79]
	SiOC glass	Spray pyrolysis	[83]
	PS-LSAO functionalised glass	Dip coating	[88]
	Hydrophobic and long-chain functionalised SiO_2_ on a polymer base layer	Dip coating	[86]
	α-cordierite	Powder sintering	[94]
	SiO_2_-Al_2_O_3_-Fe_2_O_3_-CaO-MgO-R_2_O	Dip coating	[102]
	ZnO-PDMS on polyimide	Dip coating/hydrothermal	[136]
Fuel cell component coatings	WO_3_ Polyvinylidene fluoride– hexafluoropropylene/ZrO_2_NPs	CGDS	[137] [138]
Thermal barriers	Li_2_O-Al_2_O_3_-SiO_2_	Crystallisation	[7]
	SiC	Plasma spray	[23]
	ZrB_2_-SiC-TiSi_2_	Plasma spray	[25]
	Al_2_O_3_-GdAlO_3_	Plasma spray	[28]
	(La_0.2_Nd_0.2_Sm_0.2_Eu_0.2_Gd_0.2_)_2_Zr_2_O_7_	Plasma spray	[30]
	β-Al_2_TiO_5_	Laser-assisted CVD	[44]
	YSZ-Al_2_O_3_	Plasma spray	[55]
	α-cordierite	Powder sintering	[94]
	SiO_2_-Al_2_O_3_-Fe_2_O_3_-CaO-MgO-R_2_O	Dip coating	[102]
Hydrophobic/anti-fouling surfaces	Crystalline Yb_2_O_3_	Plasma spray	[29]
	Zirconium(IV) Propoxide/Colloidal SiO_2_/methyltrimethoxysilane glass–ceramic	Sol–gel	[35]
	TiO_2_ with nitrogen plasma treatment	Sol–gel	[40]
	Zirconium epoxy-ceramic	Droplet coating	[60]
	Al_2_O_3_/ZnO phosphate ceramic reinforced with TiO_2_ nanoparticles	Powder	[66]
	Ti_3_C_2_T_x_ MXene nanosheet	Dip coating	[73]
	PFOTES-functionalised SiO_2_ nanoparticles	Sol–gel	[74]
	PFOTES-functionalised BaAl_2_Si_2_O_8_	Wet spray	[75]
	TiO_2_-functionalised PDMS	Wet spray	[76]
	SiO_2_ nanoparticles/ceramic–polymer matrix	Wet spray	[77]
	PS-LSAO-functionalised glass	Dip coating	[88]
	SiO_2_	Bar coating	[84]
	Hydrophobic and long-chain-functionalised SiO_2_ on a polymer base layer	Dip coating	[86]
	DDS functionalised SiO_2_ nanoparticles	Wet spray	[85]
	HDMS functionalised SiO_2_ nanoparticles in organosilica binder	Dip coating	[87]
	TiO_2_	Dip coating	[132]
	ZnO-PTFE composites	Sputtering	[133]
	ZnO-PDMS on polyimide	Dip coating/hydrothermal	[136]
	Double layer SiO_2_ hybrid film	Dip coating	[139]
	Hydrophilic SiO_2_-MgF_2_	Sol–gel	[140]
	SiO_2_	CGDS	[141]
Anti-reflective/transparent coating for solar cells	SiO_2_, ZnO, Al-doped ZnO, or Al_2_O_3_ coating on TiO_2_/Ag/TiO_2_ stacks for IR reflectivity	Sol–gel	[39]
	TiO_2_ with nitrogen plasma treatment	Sol–gel	[40]
	Y/Sialon	Pulsed laser deposition	[57]
	TiO_2_/SiO_2_ multilayers	Sputtering, ALD	[58,142]
	Zr-oxide doped TiO_2_/SiO_2_ multilayers	Sputtering	[59]
	Al_2_O_3_/SiO_2_	ALD	[63]
	Polymer ceramic SiO_2_	Blade coating	[64]
	Polycrystalline Y_2_O_3_ on YSZ ceramic substrates	Electron beam PVD	[68]
	MgO-ZnO	Dip coating	[69]
	Ti_3_C_2_T_x_ MXene nanosheet	Dip coating	[73]
	PFOTES-functionalised SiO_2_ nanoparticles	Sol–gel	[74]
	TiO_2_-functionalised PDMS	Wet spray	[76]
	SiO_2_ nanoparticles in ceramic–polymer matrix	Wet spray	[77]
	Epoxy-modified SiO_2_ in polysiloxane	Brush coating	[78]
	ZnO	Sputtering	[79,143]
	TiO_2_	Sputtering	[79,144]
	DDS-functionalised SiO_2_ nanoparticles	Wet spray	[85]
	Hydrophobic and long-chain-functionalised SiO_2_ on a polymer base layer	Dip coating	[86]
	HDMS-functionalised SiO_2_ nanoparticles in organosilica binder	Dip coating	[87]
	SrO-Bi_2_O_3_-B_2_O_3_	Melt quenching	[95]
	SiO_2_ nanoplates in cellulose	Sol–gel	[123]
	TiO_2_	Dip coating	[132]
	ZnO-PTFE composites	Sputtering	[133]
	Double-layer SiO_2_ hybrid film	Dip coating	[139]
	Hydrophilic SiO_2_-MgF_2_	Sol–gel	[140]
	Al-ZnO	Sputtering	[143]
	Nanostructured Al_2_O_3_ multilayers	Electron beam evaporation/sputtering	[145]
	B_2_O_5_ doped TiO_2_	Sol–gel	[146]
	*a*-Ta_2_O_5_	Sputtering	[147,148]
	Ta_2_O_5_/SiO_2_ multilayers	Sputtering	[148]
	Al_2_O_3_/Parylene-C alternating layers	ALD/CVD	[149]
	SiO_2_	Sol–gel	[150,151,152]
	Textured PDMS	CVD, spin coating, etching	[153,154,155,156]
Photonics	SiO_2_	Sol–gel	[18]
	Doped oxyfluoride glass–ceramics	Sol–gel	[36,37,38,54]
	SiO_2_, ZnO, Al doped ZnO, or Al_2_O_3_ coating on TiO_2_/Ag/TiO_2_ stacks for IR reflectivity	Sol–gel	[39]
	TiO_2_ with nitrogen plasma treatment	Sol–gel	[40]
	Polycrystalline Y_2_O_3_ on YSZ ceramic substrates	Electron beam PVD	[68]
	MgO-ZnO	Dip coating	[69]
	TiO_2_-functionalised PDMS	Spray	[76]
	PS-LSAO-functionalised glass	Dip coating	[88]
	SiO_2_-based film	Bar coating	[84]
	Double-layer SiO_2_ hybrid film	Dip coating	[139]
	TiO_2_/SiO_2_ multilayers	ALD	[142]
Oxidation barriers	Al_2_O_3_-TiO_2_	Plasma spray	[26]
	Al_2_O_3_-GdAlO_3_	Plasma spray	[28]
	MoSi_2_	Hot dip plating	[46]
	Al_2_O_3_/SiO_2_	ALD	[63]
	Y_2_SiO_5_/Y_2_O_3_-Al_2_O_3_-SiO_2_	Pulsed arc discharge/hot dipping	[90]
	SiO_2_-Na_2_O-Al_2_O_3_-K_2_O-MgO-CaO-BaO	Melt quench/spray	[91]
	Y_2_Ti_2_O_7_-SiO_2_	Melt casting	[101]
	Gradient TiO_x_-Al_2_O_3_	Thermal oxidation	[121]
	Al_2_O_3_/Parylene-C alternating layers	ALD/CVD	[149]
	MoSi_2_-SiO_2_-SiC	CGDS	[157]

**Table 4:** Key characteristics of protective coatings for photovoltaics.

Layer Material	Thickness/nm	Transparency/%		Reflectivity/%		Self- Cleaning	Contact Angle/°	Refs.
		400 nm	600 nm	400 nm	600 nm			
SiO_2_ on TiO_2_/Ag/TiO_2_	2.7 × 10^2^	>60	~80	~10	<10	Not specified	Not specified	[39]
TiO_2_ + N_2_ Plasma	~6.3 × 10^1^–9.5 × 10^1^	~90	92–95	Not specified		MB degradation	Not specified	[40] *
Y/Sialon	~1.8 × 10^1^–3.2 × 10^2^	~50	~90	Not specified		Not specified	Not specified	[57]
TiO_2_/SiO_2_	2.6 × 10^2^	80–92	92–97	3	1	Not specified	Not specified	[58] *
	~2.0 × 10^2^	>90 at 525 nm		~20	<5	Not specified	Not specified	[142] *
ZrO-doped TiO_2_/SiO_2_	2.5 × 10^2^	~88–92	88–92	<10	<5	Not specified	Not specified	[59] *
TiO_2_	<1.0 × 10^2^	~75–85	~75–85	~15–35	~10–30	Not specified	Not specified	[79]
	Particle size 2.5 × 10^1^–1.0 × 10^2^	Not specified		Not specified		MB degradation	<30	[132]
	2.8 × 10^1^–3.0 × 10^1^	Not specified		Not specified		Not specified	55–70	[144]
ZnO	3.6 × 10^1^	~70	~90	Not specified		Not specified	Not specified	[143]
	<1.0 × 10^2^	~75	~75–85	~10–25	~10–20	Not specified	Not specified	[79]
Al_2_O_3_/SiO_2_	9.4 × 10^1^	Not specified		~30	<10	Not specified	Not specified	[63] *
SiO_2_	7.0 × 10^1^–2.5 × 10^3^	~85	~90	Not specified		Not specified	88	[64]
	7.5 × 10^1^–1.4 × 10^2^	89–95	96–99	Not specified		Not specified	Not specified	[151]
	~7.7 × 10^1^	93–95	94–96	5–6	2	Not specified	Not specified	[152] *
Y_2_O_3_ on YSZ	5.0 × 10^2^–1.5 × 10^3^	~20–30	40–60	Not specified		Not specified	Not specified	[68]
		(better than substrate)						
MgO-ZnO	Not specified	(absorbance <0.5 at 400 nm)		Not specified		Not specified	Not specified	[69]
Ti_3_C_2_T_x_ MXene	~4.0 × 10^1^	50–65	71–77	Not specified		Contaminant removal	25 (glycerol sliding angle)	[73]
PFOTES/SiO_2_ NPs	3.0 × 10^4^	>80	>80	Not specified		Contaminant removal	105	[74]
		(10 days stirring)						
TiO_2_/PDMS	~5.0 × 10^1^–5.0 × 10^2^	65–80	80–85	Not specified		MB degradation, Contaminant removal	>130–>160 (recoverable)	[76]
SiO_2_ NPs in ceramic- polymer matrix	Not specified	~90	~90	Not specified		Not specified	115–>140	[77]
Epoxy-modified SiO_2_ polysiloxane	2.0 × 10^5^	~90	~90	Not specified		Not specified	Not specified	[78]
DDS/SiO_2_ NPs	1.8 × 10^5^	~30–70	~35–80	Not specified		Not specified	~160	[85]
		(thickness dependent)						
Hydrophobic SiO_2_	Not specified	91–95	92–95	Not specified		Contaminant removal	>150	[86] *
HDMS/SiO_2_ NPs in Organosilica binder	1.1 × 10^2^	95–97	96–100	Not specified		Contaminant removal	~160	[87] *
SrO-Bi_2_O_3_-B_2_O_3_ glass–ceramic	Particle size 1.0 × 10^1^–3.0 × 10^1^	0	~70	Not specified		Indicator ink and MB degradation	63	[95]
SiO_2_ nanoplates in cellulose	Ave. plate 5.4 × 10^2^	~75–80	~75–80	Not specified		Not specified	71–91	[123]
ZnO-PTFE	1.5 × 10^2^	~70–90	~85–90	Not specified		Not specified	Up to 110	[133]
Double-layer SiO_2_	~2.2 × 10^2^	~94–98	~95–98	Not specified		Contaminant removal	108–142	[139] *
SiO_2_-MgF_2_	6.9 × 10^1^–1.3 × 10^2^	87–94	92–98	<4	<3	Not specified	<6	[140]
Al-ZnO	3.6 × 10^1^–4.0 × 10^1^	~80	~80	Not specified		Not specified	109	[143,144]
Al_2_O_3_ multilayers	<1.0 × 10^2^	95–97	95–99	<20	<5	Not specified	Not specified	[145] *
B_2_O_5_ doped TiO_2_	4.7–2.3 × 10^2^	~75–100	80–100	Not specified		Not specified	16–75	[146]
a-Ta_2_O_5_	1.0 × 10^2^–1.1 × 10^2^	~72–88	~66–76	~35–48	<2	Not specified	Not specified	[147,148]
Ta_2_O_5_/SiO_2_	1.7 × 10^2^	~88	~92	5	<5	Not specified	Not specified	[148]
Al_2_O_3_/Parylene-C	1.6 × 10^3^	Visual only		Not specified		Not specified	Not specified	[149]
Textured PDMS	1.0 × 10^4^–7.0 × 10^4^	89–91	90–92	9–10	~8	Not specified	Not specified	[153]
	Not specified	Not specified		<1	<1	Not specified	140	[154]
	2.5 × 10^5^	~80–92	~80–92	~3–5	~3–5	Not specified	~126–155	[155]
	Not specified	~85–98	90	Not specified		Not specified	138	[156]

## Data Availability

No new data were created or analyzed in this study. Data sharing is not applicable to this article.

## Abbreviations

APS	Atmospheric Plasma Spraying
AYZ	Al_2_O_3_-Y_2_O_3_-ZrO_2_
CBS	CaO-B_2_O_3_-SiO_2_
CGDS	Cold Gas Dynamic Spraying
CMAS	Calcium Magnesium Aluminosilicate
CTE	Coefficient of Thermal Expansion
CVD	Chemical Vapour Deposition
DDS	Dimethyldichlorosilane
DSSC	Dye Sensitised Solar Cell
EIS	Electrochemical Impedance Spectroscopy
HMDS	Hexamethyldisilazane
LSAO	Lanthanide-doped Strontium Aluminium Oxide
MB	Methylene Blue
MCVD	Modified Chemical Vapour Deposition
PCE	Power Conversion Efficiency
PDC	Polymer Derived Ceramic
PDDA	Poly(diallyldimethylammonium)
PDMS	Polydimethylsiloxane
PECVD	Plasma Enhanced Chemical Vapour Deposition
PFOTES	1H,1H,2H,2H-perfluorooctyltriethoxysilane
PHPS	Perhydropolysilazane
PS	Polystyrene
PSC	Perovskite Solar Cell
PVD	Physical Vapour Deposition
RH	Relative Humidity
RTIC	Room-Temperature Impact Consolidation
Sialon	Silicon/Aluminium/Oxygen/Nitrogen mix
SOPS	Solid Omniphobic Slippery
YSZ	Yttria-Stabilised Zirconia
